# Two New Lactam Derivatives from *Micromelum falcatum* (Lour.) Tan. with Brine Shrimp Larvae Toxicity

**DOI:** 10.3390/molecules28207157

**Published:** 2023-10-18

**Authors:** Bin Liu, Xiaobao Jin, Xiaohong Chen, Xin Wang, Wenbo Zhang, Xiongming Luo

**Affiliations:** 1 School of Life Sciences and Biopharmaceutics, Guangdong Pharmaceutical University, Guangzhou 510006, China; vert2222@163.com (B.L.); jinxf2001@163.com (X.J.); cxh2sky@163.com (X.C.); 13245481978@163.com (W.Z.); 2 Guangdong Provincial Key Laboratory of Pharmaceutical Bioactive Substances, Guangdong Pharmaceutical University, Guangzhou 510006, China; 3 School of Basic Medical Sciences, Guangdong Pharmaceutical University, Guangzhou 510006, China; wangx357@mail2.sysu.edu.cn

**Keywords:** *Micromelum falcatum* (Lour.) Tan., lactam derivatives, brine shrimp larvae

## Abstract

Chemical investigation of the stems of *Micromelum falcatum* (Lour.) Tan. led to the isolation of two new lactam derivatives, named 3-(hydroxy(10-hydroxyphenyl)methyl)-4-(16-hydroxyphenyl)-1-methylpyrrolidin-2-one (**1**) and 3-(hydroxy(10-hydroxy-9-methoxyphenyl)methyl)-4-(16-hydroxyphenyl)-1-methylpyrrolidin-2-one (**2**), along with five known compounds, trans-4-hydroxycinnamic acid (**3**), 4-hydroxybenzaldehyde (**4**), m-hydroxybenzoic acid (**5**), p-hydroxybenzoic acid (**6**), and gallic acid (**7**). Their structures were determined on the basis of spectroscopic studies, including nuclear magnetic resonance (NMR) spectrum, mass spectrometry (MS) data, ultraviolet (UV) spectrum, infrared (IR) data, and comparison with the literature. All compounds were evaluated for toxicity against brine shrimp larvae and cytotoxicity to HeLa and HepG-2 cells. Compounds **1**–**2** exhibited moderate brine shrimp larvae toxicity with an LC_50_ value of 50.6 and 121.8 μg mL^−1^, respectively.

## 1. Introduction

*Micromelum falcatum* (Lour.) Tan. (*M. falcatum*) is a mangrove-associated plant of the *Rutaceae* family and the genus *Micromelum*. Its flowers have unpleasant aromas. Fruits can be green, orange, or red due to their varying degrees of maturity. *M. falcatum* is mainly distributed in the southwest of Guangdong Province, Hainan Province, Guangxi Province, and the southeast of Yunnan Province [1,2,3,4,5,6]. It is also found in Vietnam, Laos, Cambodia, and Thailand [3]. *Micromelum* is a genus of the *Rutaceae* family, including approximately 10 species, most of which have medicinal values and are usually used to treat various diseases in traditional folk medicine [1,3,4]. For example, people use the leaves, stems, and roots of *Micromelum integerrimum* as medicine to dissipate blood stasis, relieve pain, and treat conditions such as stomachaches and rheumatic bone pain, among others [1,6]. *M. falcatum* is used to treat injuries, such as traumatic injury, snakebite, and rheumatism [7]. Since the 1960s, many researchers have studied the chemical composition of this genus of plants, and more than 100 compounds have been isolated and identified from the extracts of the roots, bark, and leaves, including coumarins, alkaloids, flavonoids, triterpenoids, and sterol, etc. [1,6,7,8,9].

Our interest in this plant was stimulated by the discovery of the potent anti-implantation alkaloid yuehchukene isolated from *M. falcatum* [10]. The dimeric indole alkaloid yuehchukene showed a strong ability to completely prevent implantation up to day 5 of pregnancy at 2 mg kg^−1^ in rats [11]. In order to search for new toxic agents, we further investigated the chemical constituents of *M. falcatum* collected from Sanya, Hainan Province. Herein, we report the structural elucidation and brine shrimp toxicity assessment of two new lactam derivatives (**1**–**2**). Additionally, five known compounds were identified through a combination of NMR, MS and comparison with the literature. These compounds were characterized as trans-4-hydroxycinnamic acid (**3**) [12], 4-hydroxybenzaldehyde (**4**) [13], m-hydroxybenzoic acid (**5**) [14], p-hydroxybenzoic acid (**6**) [15], and gallic acid (**7**) [16]. The isolated compounds **1** and **2** exhibited pronounced to moderate toxic activity against the brine shrimp larvae with LC_50_ values of 50.6 and 121.8 μg mL^−1^_,_ respectively. Cytotoxicity was evaluated by determining the IC_50_ value against two human cancer cells (HeLa and HepG-2) using the CCK-8 assay. None of the compounds exhibited good cytotoxicity.

## 2. Results

The ethyl alcohol (EtOH) extract from the stems of *M. falcatum* was partitioned with n-hexane and ethyl acetate (AcOEt), as described in Section 4. The AcOEt extract was subjected to silica gel column chromatography, Sephadex LH-20, and semi-preparative HPLC to yield two new lactam derivatives, **1**–**2**, in addition to the five known compounds: trans-4-hydroxycinnamic acid (**3**), 4-hydroxybenzaldehyde (**4**), m-hydroxybenzoic acid (**5**), p-hydroxybenzoic acid (**6**), and gallic acid (**7**). The structures of compounds **1**–**7** were elucidated through the analysis of spectroscopic data, including MS, UV, IR, and NMR spectra, as well as by comparison with previously published data.

**Figure 1:**
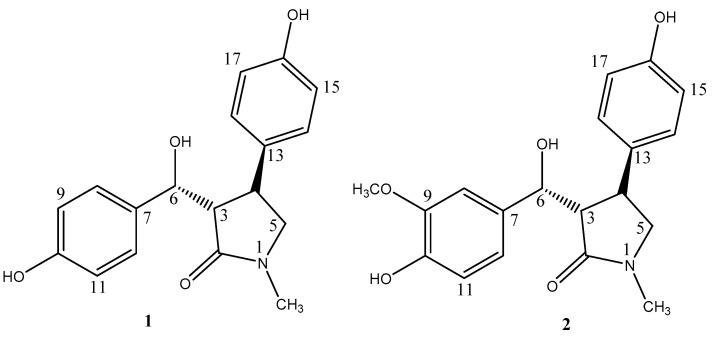
Structures of compounds **1**–**2**.

**Figure 2:**
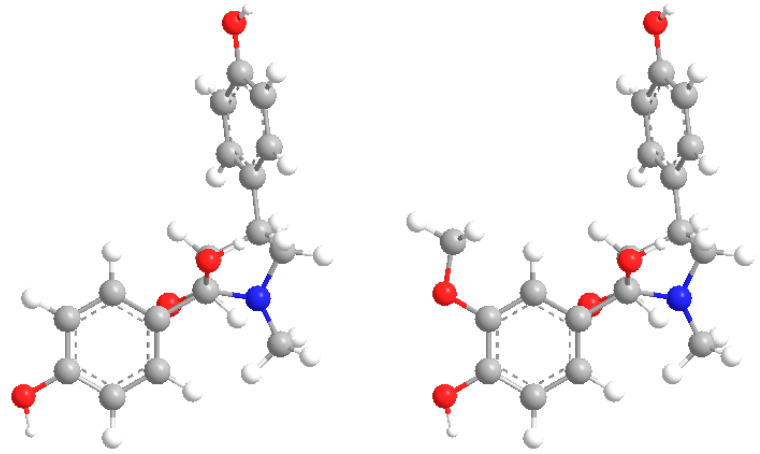
Three-dimensional structures of compounds **1**–**2**.

Compound **1** was obtained as a yellow oil with [α${]}_{D}^{20}$-12.2 (c = 0.1, MeOH). The molecular formula of **1** was determined to be C_18_H_19_O_4_N via high-resolution electrospray ionization mass spectrometry (HR-ESI-MS) *m*/*z*: 336.1216 (calcd for C_18_H_19_O_4_NNa^+^[M + Na]^+^, 336.1206). The UV spectrum showed absorption bands at λmax 228 and 276 nm. Its IR spectrum revealed absorption bands at v_max_ = 3262, 1651, 1614, and 1516 cm^−1^, which indicated the presence of hydroxyl, amide carbonyl, and aromatic groups. The ^1^H-NMR spectrum of **1** (Table 1) exhibited two aromatic A_2_B_2_ systems (*δ*_H_ 7.13 (2H, d, J = 8.5 Hz), 6.68 (2H, d, J = 8.5 Hz), and *δ*_H_ 6.64 (2H, d, J = 8.5 Hz), 6.88 (2H, d, J = 8.5 Hz)), one methyl (*δ*_H_ 2.80 (3H, s, N-CH_3_)), and one methylene (*δ*_H_ 3.15 (2H, d, J = 2.5 Hz)). The ^13^C-NMR and DEPT spectra of **1** showed the presence of one methyl group (*δ*_C_ 29.7 (N-CH_3_)), one methylene (*δ*_C_ 57.4), eleven methines (*δ*_C_ 39.7, 59.0, 75.3, 115.8, 115.8, 116.4, 116.4, 129.1, 129.1, 129.1, and 129.1), and five quaternary carbons (*δ*_C_ 132.6, 135.7, 157.1, 158.3, and 176.9). These above data were similar to those for lepiotins A and B [17] and suggested that **1** has γ-lactam and two aromatic rings (Figure 1 and Figure 2).

**Figure 3:**
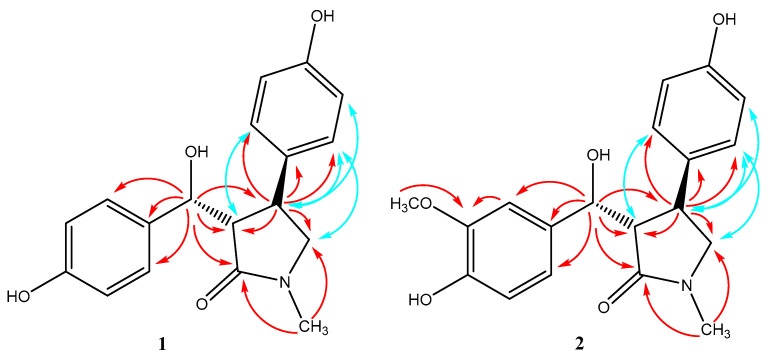
Key HMBC (red) and NOESY (blue) correlations of compounds **1**–**2**.

In the HMBC spectrum, the correlations between *δ*_H_ 2.80 (s, N-CH_3_) and *δ*_C_ 176.9 (C-2), 57.4 (C-5), between *δ*_H_ 2.93 (1H, dd, J = 6.0, 5.5 Hz, H-3) and *δ*_C_ 176.9 (C-2), 39.7 (C-4), and between *δ*_H_ 3.15 (2H, d, 2.5 Hz, H-5) and *δ*_C_ 176.9 (C-2), 39.7 (C-4) indicated the presence of a γ-lactam. Meanwhile, the HMBC correlations of *δ*_H_ 4.95 (1H, d, 6.0 Hz, H-6) with *δ*_C_ 59.0 (C-3), 39.7 (C-4), 132.6 (C-7), and 129.1 (C-8, 12) revealed one aromatic ring connecting with the γ-lactam skeleton through C-6. The HMBC correlations of *δ*_H_ 3.25 (1H, m, H-4) with *δ*_C_ 59.0 (C-3), 57.4 (C-5), 135.7 (C-13), and 129.1 (C-14, 18) revealed the other aromatic ring locating at C-4 of the γ-lactam skeleton (Figure 3). The ^13^C chemical shifts of C-6, 10, and 16 at *δ*_C_ 75.3, 158.3, and 157.1, respectively, indicated hydroxyl groups as their neighboring substituents, which was also confirmed by the HR-ESI-MS data. Based on these data, the structure of **1** was concluded and named 3-(hydroxy(10-hydroxyphenyl)methyl)-4-(16-hydroxyphenyl)-1-methylpyrrolidin-2-one.

The relative configuration of compound **1** was deduced from the results of the NOESY experiment. The cross peaks between H-3 at *δ*_H_ 2.93 and H-18 at *δ*_H_ 6.88, H-4 at *δ*_H_ 3.25 and H-14 at *δ*_H_ 6.88, H-5 at *δ*_H_ 3.15 and H-14 at *δ*_H_ 6.88, H-4 at *δ*_H_ 3.25 and H-15 at *δ*_H_ 6.64 were observed, and the trans configurations between H-3 and H-4 could be established.

Compound **2** was obtained as a yellow oil with [α${]}_{D}^{20}$-12.8 (c = 0.1, MeOH). The molecular formula of **2** was established as C_19_H_21_NO_5_ via HR-ESI-MS *m*/*z*: 366.1317 (calcd for C_19_H_21_NO_5_Na^+^ [M + Na]^+^, 366.1312).The ^1^H- and ^13^C-NMR spectra of **2** (Table 1) were similar to those of **1,** with the only difference being an aromatic proton (*δ*_H_ 6.68 (1H, d, 8.5 Hz, H-9)) for **1**, substituted by a methoxy group (*δ*_H_ 3.75 (3H, s), *δ*_C_ 56.4) for 2 (Figure 1). This suggested that compound **2** was also a γ-lactam with two aromatic rings, which was proved by the HMBC spectrum, showing correlations of *δ*_H_ 2.84 (s, N-CH_3_) with C-2/C-5, *δ*_H_ 4.91 (1H, d, 7.0 Hz, H-6) with C-2/C-3/C-4/C-7/C-8/C-12, and *δ*_H_ 3.21 (1H, m, H-4) with C-3/C-5/C-13/C-14/C-18 (Figure 3). So, the structure of **2** was assigned as 3-(hydroxy(10-hydroxy-9-methoxyphenyl)methyl)-4-(16-hydroxyphenyl)-1-methylpyrrolidin-2-one.

The relative configuration of compound **2** was also deduced from the results of the NOESY experiment. The cross peaks between H-3 at *δ*_H_ 2.97 and H-18 at *δ*_H_ 6.86, H-4 at *δ*_H_ 3.21 and H-14 at *δ*_H_ 6.86, H-5 at *δ*_H_ 3.22 and H-14 at *δ*_H_ 6.86, and H-4 at *δ*_H_ 3.21 and H-15 at *δ*_H_ 6.63 were observed. These signals demonstrated that compounds **2** and **1** had the same relative configurations.

The toxic activity of all isolated compounds from *M. falcatum* was evaluated against brine shrimp larvae using a 96-well plate assay. Compounds **1**–**2** had moderate activity and exhibited LC_50_ values of 50.6 and 121.8 μg mL^−1^, respectively (Table 2).

In addition, cytotoxicity was evaluated based on the IC_50_ value against two human cancer cells (Hela and HepG-2) using the CCK-8 assay. None of the compounds showed good cytotoxicity.

## 3. Discussion

Lactams are a class of compounds with the cyclic structure of R_1_-CONH-R_2_ (the R group is generally a hydrocarbon group). The lactam rings have four to seven members, which can be synthesized using methods, such as acylation reactions, aminolysis reactions of amides, and cyclization reactions. The lactam skeleton is widely found in many natural products and synthetic molecules [18,19,20,21]. For example, lajollamycin, a natural nitro-tetraene spiro-β-lactone-γ-lactam antibiotic isolated from a strain of Streptomyces nodosus, exhibits antimicrobial activity against both drug-sensitive and drug-resistant microorganisms, and it shows cytotoxicity to the B16-F10 cell line with an EC_50_ of 9.6 µM [19]. Rolipram, a γ-lactam synthetic compound, was developed as an inhibitor of phosphodiesterase iv(pde-4), which is used for depression, with better antidepressant effects and tolerance than tricyclic antidepressants [22]. In recent decades, chemists have made many efforts to achieve the precise construction of chiral lactams using organic asymmetric catalysis and enzyme catalysis. Lactams have extensive applications in various fields, including pharmaceuticals, polymer materials, and pesticides [20,23]. Additionally, 2-azabicyclo[2.2.1]hept-5-en-3-one (γ-lactam) is also a key chiral synthon in the synthesis of the antiviral drugs carbovir and abacavir [24]. The two new lactam derivatives reported in this study are γ-lactam structures with substituents at positions 3 and 4, which theoretically have the potential to be developed.

In 2014, five phenethyl cinnamides isolated from *M. falcatum* [6], all of which have a core of micrometal C, were found in which micrometam C significantly decreased the elevation of reactive oxygen species levels, increase catalase, glutathione, and superoxide dismutase levels, and reduced the inflammation-associated migration of immune cells [25]. In terms of biosynthetic pathways, two new lactam derivatives(γ-lactams) **1**–**2** may be synthesized from these five phenethyl cinnamides via the cyclization of phenethyl cinnamides into a ring.

## 4. Materials and Methods

### 4.1. General Experimental Procedures

LC-ESI-MS was recorded on a SHIMADZU PR-LC MS-2020 LC/MS/MS mass spectrometer. HRESI-MS was performed using a VG Auto Spec-3000 MS spectrometer and an Agilent G6540A UHD Accurate-Mass Q-TOF LC/MS with a 1260 HPLC System (Agilent Technologies, Santa Clara, CA, USA). NMR spectra were recorded on a Bruker DRX-500MHz and Bruker Avance Ⅲ HD 400 MHz spectrometer with tetramethylsilane(TMS) as the internal standard (Bruker, Billerica, MA, USA). Optical rotation and IR spectra were measured on a Polaptronic-HNQW5 high-resolution polarimeter and a SHIMADZU IRPrestige-21 Fourier-Transform Infrared Spectrometer. Silica gel (200–300 mesh; Qingdao Haiyang Chemical Plant, Qingdao, China) and Sephadex LH-20 (GE Healthcare, Chicago, IL, USA) were used for the column chromatography. Thin-layer chromatography (TLC) was performed on pre-coated silica gel G plates (300–400 mesh, Qingdao Haiyang Chemical Plant, Qingdao, China), and spots were visualized by spraying the plates with a 50% H_2_SO_4_ solution, followed by heating. A Waters e2695 HPLC system (Phenomenex Luna 5u C18(2) 100 A, 250 nm × 4.60 mm) was equipped with a Waters 2998 photodiode array detector. Semi-preparative RP-HPLC was performed on ODS columns (YMC-Pack ODS-5-A, 250 mm × 10 mm, i.d., 5 μm, YMC) using a CH_3_OH–H_2_O solvent system as the eluent. Deuterated dimethyl sulfoxide-d_6_ (DMSO-d_6_), Methanol-d_4_ (CD_3_OD), and chloroform-d (CDCl_3_) were supplied by Cambridge Isotope Laboratories, Inc. (Andover, MA, USA). DMEM and fetal bovine serum were supplied by Gibco Inc. (New York, NY, USA). The CCK-8 reagent was obtained from Beyotime Biotechnology (Shanghai, China). The cytotoxicity assay data were read using a microplate reader (BioTek EL808, Highland Park, VT, USA). By means of HPLC-MS, ^1^H-NMR, and ESI-MS, the purity of all compounds was investigated, and the degree of purity of all the tested compounds was >95%. All other reagents and solvents used were of reagent or HPLC analytical grade.

### 4.2. Plant Material

*Micromelum falcatum* (Lour.) Tan. collected from Sanya, Hainan Province, China, in January 2018, was authenticated by Prof. Jun Wu, Guangdong Medical University, and a voucher specimen was deposited at the Guangdong Provincial Key Laboratory of Pharmaceutical Bioactive Substances (No. MA-20180916-02).

### 4.3. Extraction and Isolation

The dried aerial parts of *Micromelum falcatum* (Lour.) Tan. were ground to a fine powder to obtain a 3.5 kg sample, which was extracted with 95% EtOH (15.0 L) three times. After the solvent was evaporated under reduced pressure, the residue (511.3 g) was extracted with n-hexane and EtOAc (4 × 2 L each). The EtOAc extract (85.0 g) was separated on silica gel (815 g, 200–300 mesh) using solvents of increasing polarity, 10–70% acetone in n-hexane, followed by 5–100% MeOH in CHCl_3_, to obtain 107 fractions. According to the TLC analysis, the fraction 15 (1.59 g) was subjected to CC (chloroform: acetone, 15:1) to yield a crude component, which was further purified using Sephadex LH-20 (MeOH) to yield **4** (15.0 mg). Fractions 23–25 (3.75 g) were subjected to CC (chloroform: acetone, 10:1) to yield fractions A-F. Fraction B was purified by Sephadex LH-20 (MeOH) to give **3** (13.7 mg) and **5** (11.3 mg), and fraction C was purified by semi-preparative HPLC (250 mm × 10 mm i.d., 5 μm, MeOH/H_2_O, 40:60, flow rate 3 mL min^−1^, UV detection at 254 and 280 nm) to yield 6.8 mg of **6** and 13.6 mg of **7**. Fractions 43–44 were combined to give a 2.6 g mixture. The mixture was fractionated on silica gel (350 g, 200–300 mesh) with chloroform-acetone (8:2), and a total of 22 sub-fractions (ca. 180 mL each) were collected and combined using TLC. After purification using Sephadex LH-20, sub-fractions 17–20 (75–375 mL) were further separated via semi-preparative HPLC (250 × 10 mm i.d., 5 μm, MeOH/H_2_O, 50:50, flow rate 3 mL min^−1^, UV detection at 254 and 280 nm) to generate 6.2 mg of **1** and 14.2 mg of **2** [26,27,28].

3-(Hydroxy(10-hydroxyphenyl)methyl)-4-(16-hydroxyphenyl)-1-methylpyrrolidin-2-one (**1**): yellow oil; [α${]}_{D}^{20}$-12.2 (c = 0.1, MeOH); UV-Vis (EtOH) λ/nm 228, 276; IR (KBr) *v*/cm^−1^:3262, 1651, 1614, 1516, 1248, 833; Positive ESI-MS *m*/*z*, observed: 649 [2M + Na]^+^ (45), 336 [M + Na]^+^ (93), 141 (100); HR-ESI-MS *m*/*z*, observed: 336.1216 (calcd for C_18_H_19_NO_4_Na^+^ [M + Na]^+^, 336.1206); ^1^H- and ^13^C-NMR data, see Table 1.

3-(Hydroxy(10-hydroxy-9-methoxyphenyl)methyl)-4-(16-hydroxyphenyl)-1-methylpyrrolidin-2-one (**2**): yellow oil; [α${]}_{D}^{20}$-12.8 (c = 0.1, MeOH); UV-Vis (EtOH) λ/nm 228, 278; IR (KBr) *v*/cm^−1^: 3363, 1653, 1603, 1514, 1269, 1250, 831; Positive ESI-MS *m*/*z*, observed: 709 [2M + Na]^+^ (28), 366 [M + Na]^+^ (100); HR-ESI-MS *m*/*z*, observed: 366.1317; (calcd for C_19_H_21_NO_5_Na^+^ [M + Na]^+^, 366.1312); ^1^H- and ^13^C-NMR data, see Table 1.

### 4.4. Brine Shrimp Larvae Lethality Assays

Brine shrimp spawns were incubated in a beaker containing seawater and cultivated for 48 h at room temperature (22–29 °C). Brine shrimp larvae flocked together on one side of the container with the assistance of a luminous source, which was readily assembled for the brine shrimp larval lethality assay. Compounds **1**–**7** were dissolved in DMSO at a concentration of 50 mg mL^−1^, then diluted in a 96-well plate with 200 µL of seawater to test at final concentrations of 5, 50, and 500 μg mL^−1^. Each test was conducted in triplicate with approximately ten larvae. After 24 h of cultivation, the brine shrimp larvae were tallied under a luminous source in 96-well plates using a magnifier. LC_50_ values for each assay were calculated using a Finney Probit analysis program on a Dell computer [29].

### 4.5. Cytotoxicity Assays

In brief, 100 µL of the cell suspension was seeded into each well of 96-well plates (6 × 10^3^ per well) and then incubated for 12–24 h to complete cell attachment. Fresh medium containing concentrations of test compounds was added to each well after removing the medium. Each concentration was tested in triplicate. After culturing for 24 h, 10 µL of the CCK-8 reagent was added to each well and incubated in a CO_2_ incubator for 1 h. The absorbance was measured at 450 nm using a microplate reader (BioTek EL808, Highland Park, VT, USA). The cytotoxicity assay data were read using Gen5 CHS V2.01 software [30].

## 5. Conclusions

The present phytochemical investigation of *M. falcatum* afforded two new lactam derivatives, 3-(hydroxy(10-hydroxyphenyl)methyl)-4-(16-hydroxyphenyl)-1-methylpyrrolidin-2-one (**1**) and 3-(hydroxy(10-hydroxy-9-methoxyphenyl)methyl)-4-(16-hydroxyphenyl)-1-methylpyrrolidin-2-one (**2**), and five known compounds, trans-4-hydroxycinnamic acid (**3**), 4-hydroxybenzaldehyde (**4**), m-hydroxybenzoic acid (**5**), p-hydroxybenzoic acid (**6**), and gallic acid (**7**). All the isolated compounds were evaluated for their brine shrimp larvae and cytotoxic activities against HeLa and HepG-2 cells. Unfortunately, no compounds showed good cytotoxicity. The brine shrimp larvae lethality bioassay of the isolated compounds **1**–**2** showed moderate activity, exhibiting LC_50_ values of 50.6 and 121.8 μg mL^−1^, respectively. After determining the relative configurations of compounds **1**–**2** through the NOESY spectrum, we attempted to determine the absolute configurations. However, the Mosher reaction did not succeed. Compounds **1**–**2** also cannot easily form crystalline states. Due to the limited weight of the separated compounds, we only tested two activities and did not screen out compounds with good activity. Future work can start from multiple directions, such as determining the absolute configuration of compounds **1**–**2** through other methods (for instance, circular dichroism calculation), separating more weight of these compounds and testing other activities, or studying their drug synthesis pathways, and so on.

## Figures and Tables

**Table 1:** NMR spectral data for **1**–**2** (500 MHz for ^1^H-NMR and 125 MHz for ^13^C-NMR in CD_3_OD).

Position	1		2	
	*δ_C_* (ppm)	*δ_H_* (ppm)	*δ_C_* (ppm)	*δ_H_* (ppm)
2	176.9	-	177.0	-
3	59.0	2.93 (1H, dd, 6.0, 5.5 Hz)	58.6	2.97 (1H, dd, 7.0, 5.5 Hz)
4	39.7	3.25 (1H, m)	40.2	3.21 (1H, m)
5	57.4	3.15 (2H, d, 2.5 Hz)	57.4	3.22 (2H, d, 2.0 Hz)
6	75.3	4.95 (1H, d, 6.0 Hz)	75.8	4.91 (1H, d, 7.0 Hz)
7	132.6	-	133.2	-
8	129.1	7.13 (1H, d, 8.5 Hz)	111.6	6.80 (1H, s)
9	115.8	6.68 (1H, d, 8.5 Hz)	148.8	-
10	158.3	-	147.5	-
11	115.8	6.68 (1H, d, 8.5 Hz)	115.6	6.70 (1H, d, 8.0 Hz)
12	129.1	7.13 (1H, d, 8.5 Hz)	121.0	6.77 (1H, d, 8.0 Hz)
13	135.7	-	135.3	-
14	129.1	6.88 (1H, d, 8.5 Hz)	129.2	6.86 (1H, d, 8.5 Hz)
15	116.4	6.64 (1H, d, 8.5 Hz)	116.3	6.63 (1H, d, 8.5 Hz)
16	157.1	-	157.1	-
17	116.4	6.64 (1H, d, 8.5 Hz)	116.3	6.63 (1H, d, 8.5 Hz)
18	129.1	6.88 (1H, d, 8.5 Hz)	129.2	6.86 (1H, d, 8.5 Hz)
1-Me	29.7	2.80 (3H, s)	29.8	2.84 (3H, s)
9-OMe		-	56.4	3.75 (3H, s)

**Table 2:** The LC_50_ values of compounds **1**–**2** against brine shrimp larvae.

Compounds	LC_50_ (Brine Shrimp) μg mL^−1^
1	50.6
2	121.8

## Data Availability

Data is contained in the article or Supplementary Materials.

## Supplementary Materials

The following supporting information can be downloaded at: https://www.mdpi.com/article/10.3390/molecules28207157/s1, Figures S1–S33 including 1H-NMR, 3C-NMR and mass figures of all isolated compounds as well as 2D NMR and IR figures for compounds 1-2.

## Sample Availability

Samples of compounds **1**–**7** are available from the authors.

## Abbreviations

The following abbreviations are used in this manuscript:

EtOAc	Ethyl acetate
EtOH	Ethyl alcohol
AcOEt	Ethyl acetate
MeOH	Methanol
IR	Infrared
NMR	Nuclear magnetic resonance
HR-ESI-MS	High resolution electrospray ionization mass spectroscopy
DEPT	Distortionless Enhancement by Polarization Transfer
HMBC	Heteronuclear multiple bond correlation
HSQC	Heteronuclear single quantum correlation
COSY	Homonuclear chemical shift Correlation Spectroscopy
NOESY	Nuclear Overhauser effect spectroscopy
ODS	Octadecyl silane
HPLC	High performance liquid chromatography
DMSO	Dimethyl sulfoxide
CCK-8	Cell Counting Kit-8
