# Supplementary material for: Two New Lactam Derivatives from Micromelum falcatum (Lour.) Tan. with Brine Shrimp Larvae Toxicity
Source: Molecules. 2023 Oct 18;28(20):7157. doi: 10.3390/molecules28207157 (PMC10608822; doi:10.3390/molecules28207157)
Supplement: Supplementary file 1 [file molecules-28-07157-s001.zip › molecules-2597466-supplementary.pdf]

Two New Lactam Derivatives from *Micromelum falcatum* (Lour.) Tan. with Brine Shrimp Larvae Toxicity

Supplementary Materials

Bin Liu [1,2], Xiaobao Jin [1,2], Xiaohong Chen [1,2], Xin Wang [1], Wenbo Zhang [1,2] and Xiongming Luo [1,2,*]

1 School of Life Sciences and Biopharmaceutics, Guangdong Pharmaceutical University, Guangzhou 510006, China;

2 Guangdong Provincial Key Laboratory of Pharmaceutical Bioactive Substances, Guangdong Pharmaceutical University, Guangzhou 510006, China;

* Correspondence: qxjun312xx@gdpu.edu.cn

List of Figures S1-S33

Figure S1. ^{1}H-NMR (500MHz, CD_3OD) spectrum of the compound **1**

Figure S2. ^{13}C-NMR (125MHz, CD_3OD) spectrum of the compound **1**

Figure S3. DEPT spectrum of the compound **1**

Figure S4.NOESY spectrum of the compound **1**

Figure S5. HSQC spectrum of the compound **1**

Figure S6. HMBC spectrum of the compound **1**

Figure S7. ESI-MS spectrum of the compound **1**

Figure S8. HR-ESI-MS spectrum of the compound **1**

Figure S9. IR spectrum of the compound **1**

Figure S10.^{1}H-NMR (500MHz, CD_3OD) spectrum of the compound **2**

Figure S11.^{13}C-NMR (125MHz, CD_3OD) spectrum of the compound **2**

Figure S12.DEPT spectrum of the compound **2**

Figure S13.NOESY spectrum of the compound **2**

Figure S14. HSQC spectrum of the compound **2**

Figure S15. HMBC spectrum of the compound **2**

Figure S16. ESI-MS spectrum of the compound **2**

Figure S17. HR-ESI-MS spectrum of the compound **2**

Figure S18. IR spectrum of the compound **2**

Figure S19.^{1}H-NMR (400MHz, DMSO-d_6) spectrum of the compound **3**

Figure S20.^{13}C-NMR (100MHz, DMSO-d_6) spectrum of the compound **3**

Figure S21. ESI-MS spectrum of the compound **3**

Figure S22. ^{1}H-NMR (400MHz, $CDCl_3$) spectrum of the compound **4**

Figure S23. ^{13}C-NMR (100MHz, $CDCl_3$) spectrum of the compound **4**

Figure S24. ESI-MS spectrum of the compound **4**

Figure S25. ^{1}H-NMR (400MHz, $CDCl_3$) spectrum of the compound **5**

Figure S26. ^{13}C-NMR (100MHz, $CDCl_3$) spectrum of the compound **5**

Figure S27. ESI-MS spectrum of the compound **5**

Figure S28. ^{1}H-NMR (400MHz, $CDCl_3$) spectrum of the compound **6**

Figure S29. ^{13}C-NMR (100MHz, $CDCl_3$) spectrum of the compound **6**

Figure S30. ESI-MS spectrum of the compound **6**

Figure S31. ^{1}H-NMR (400MHz, DMSO-d6) spectrum of the compound **7**

Figure S32. ^{13}C-NMR (100MHz, DMSO-d6) spectrum of the compound **7**

Figure S33. ESI-MS spectrum of the compound **7**

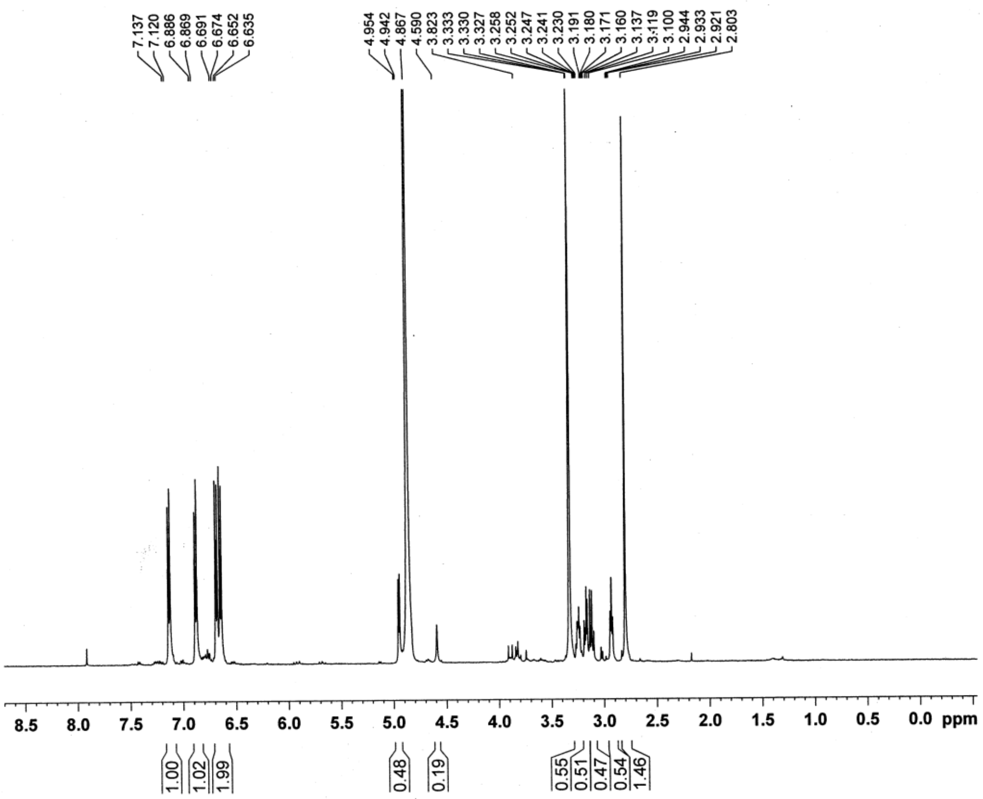

Figure S1. ^{1}H-NMR (500MHz, CD_3OD) spectrum of the compound **1**

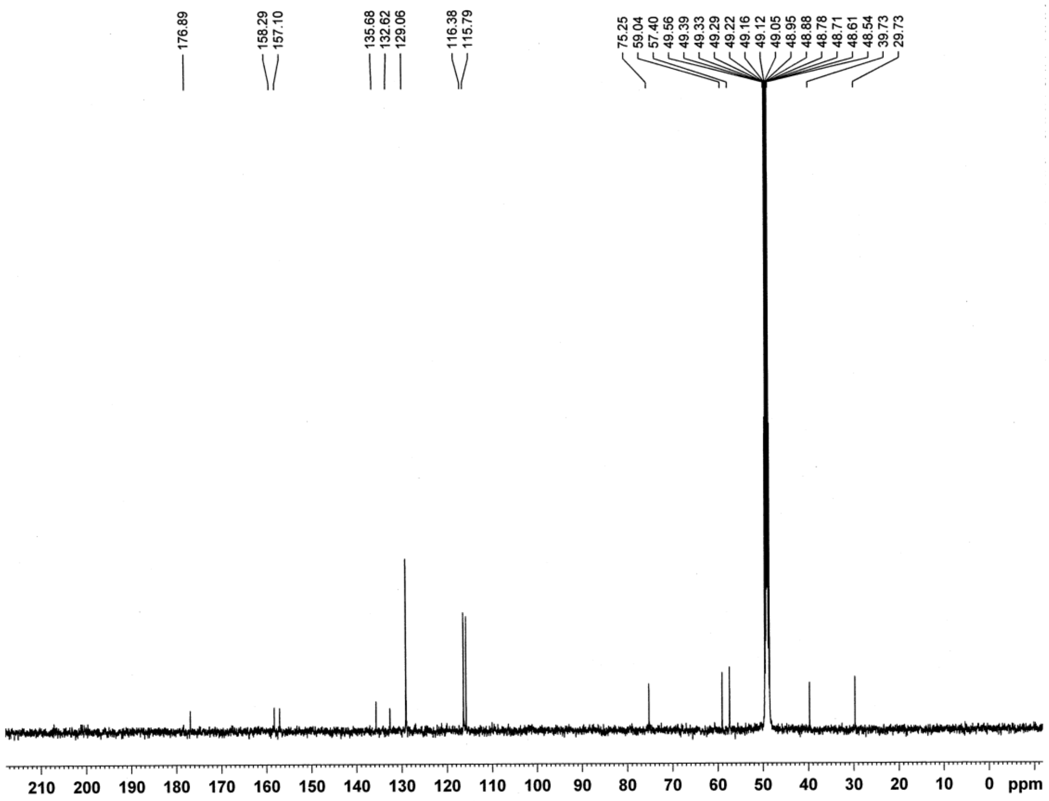

Figure S2. ^{13}C-NMR (125MHz, CD_3OD) spectrum of the compound **1**

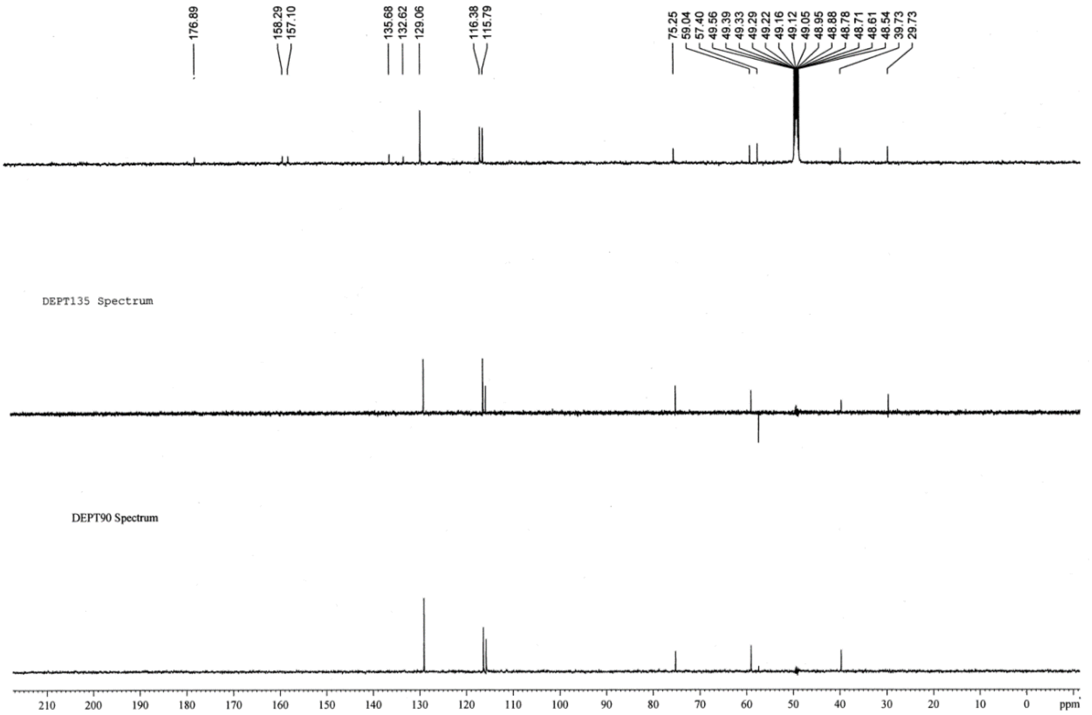

Figure S3. DEPT spectrum of the compound **1**

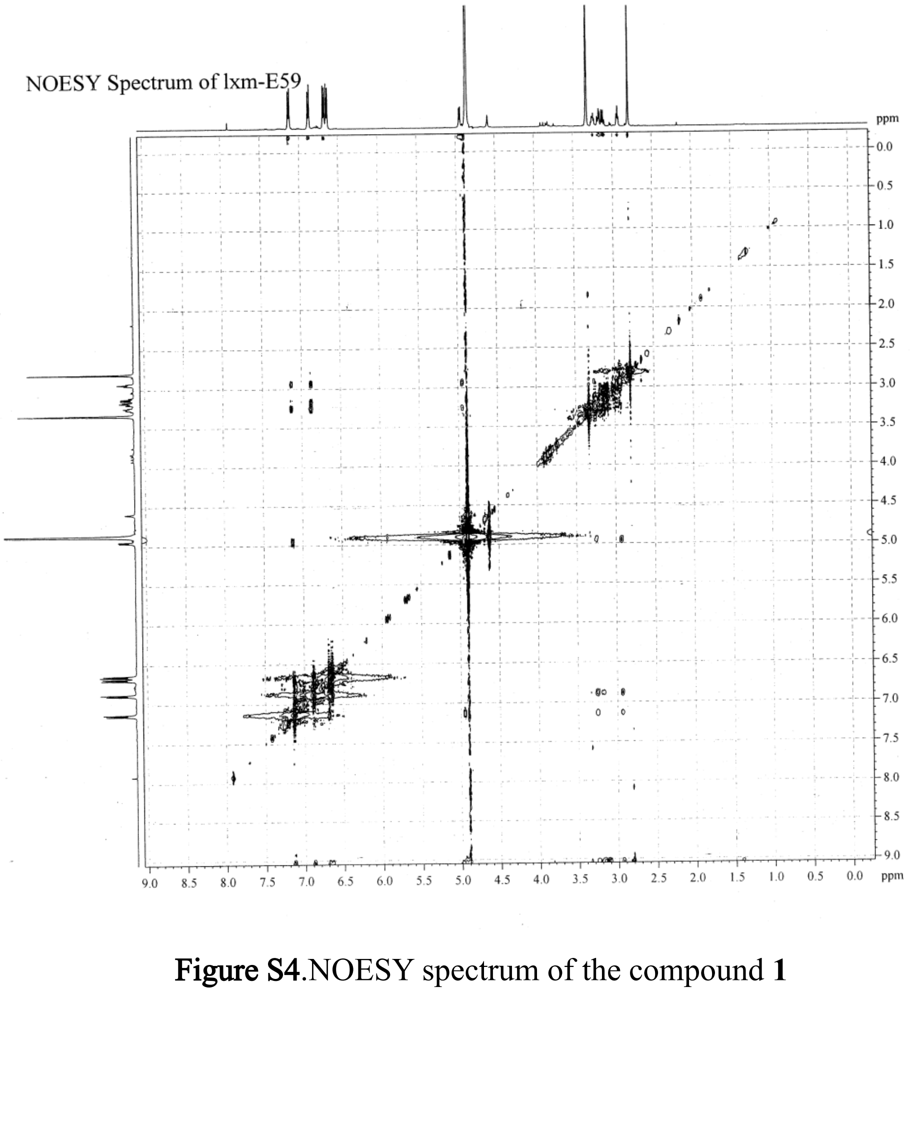

Figure S4.NOESY spectrum of the compound **1**

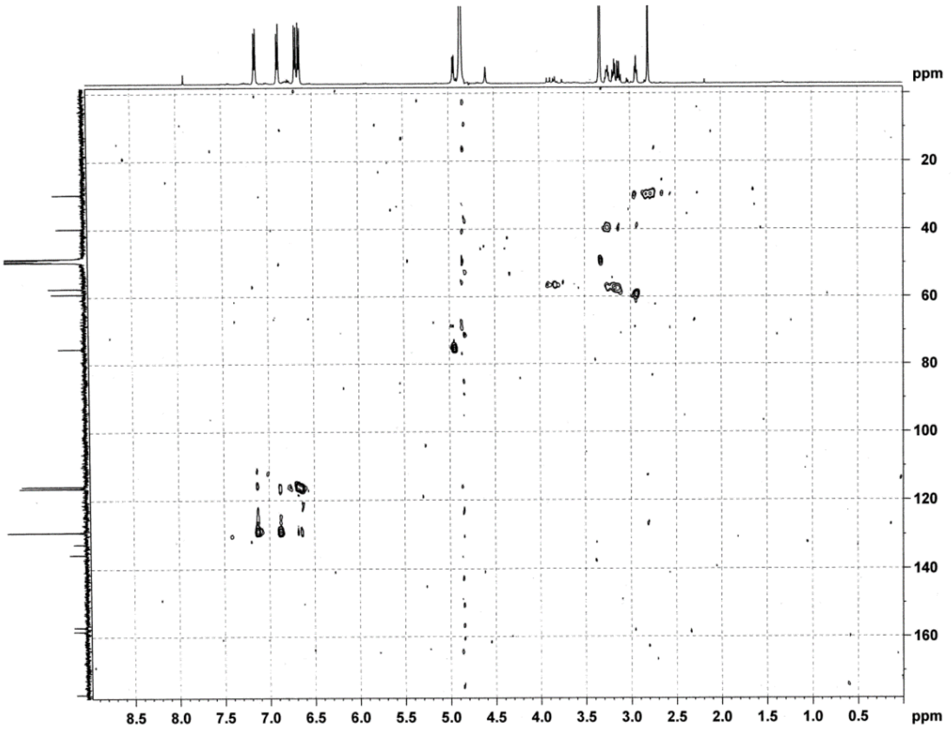

Figure S5. HSQC spectrum of the compound **1**

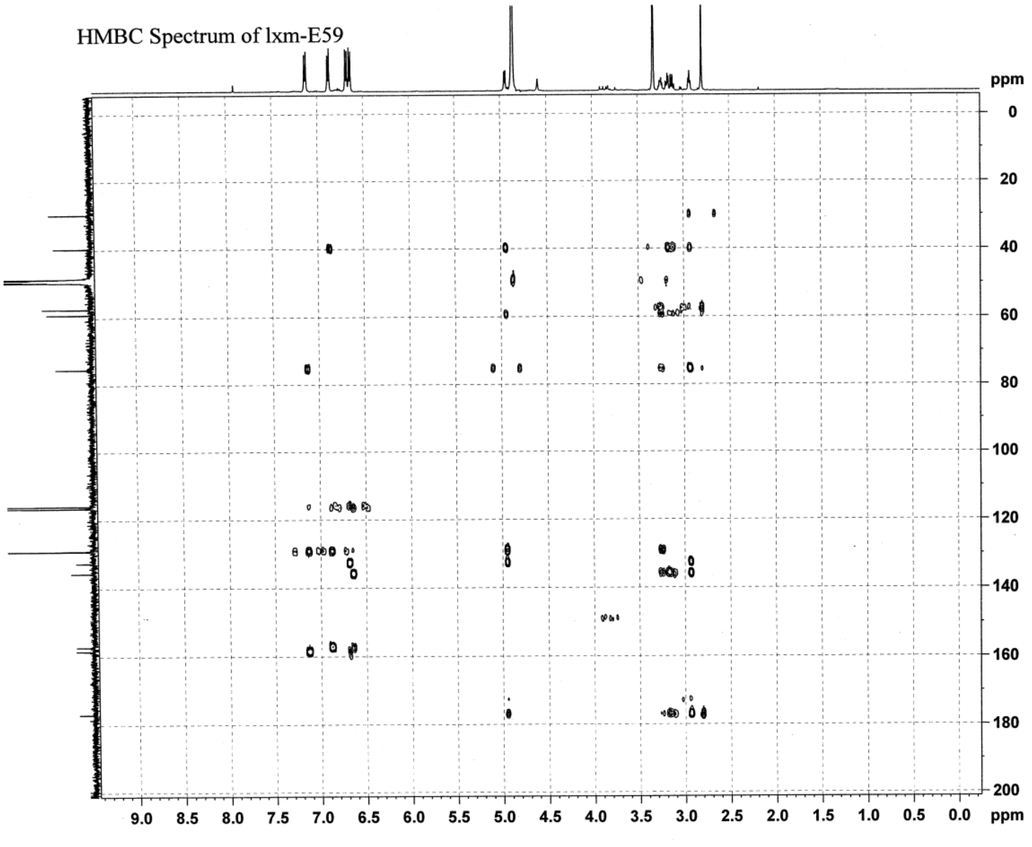

Figure S6. HMBC spectrum of the compound **1**

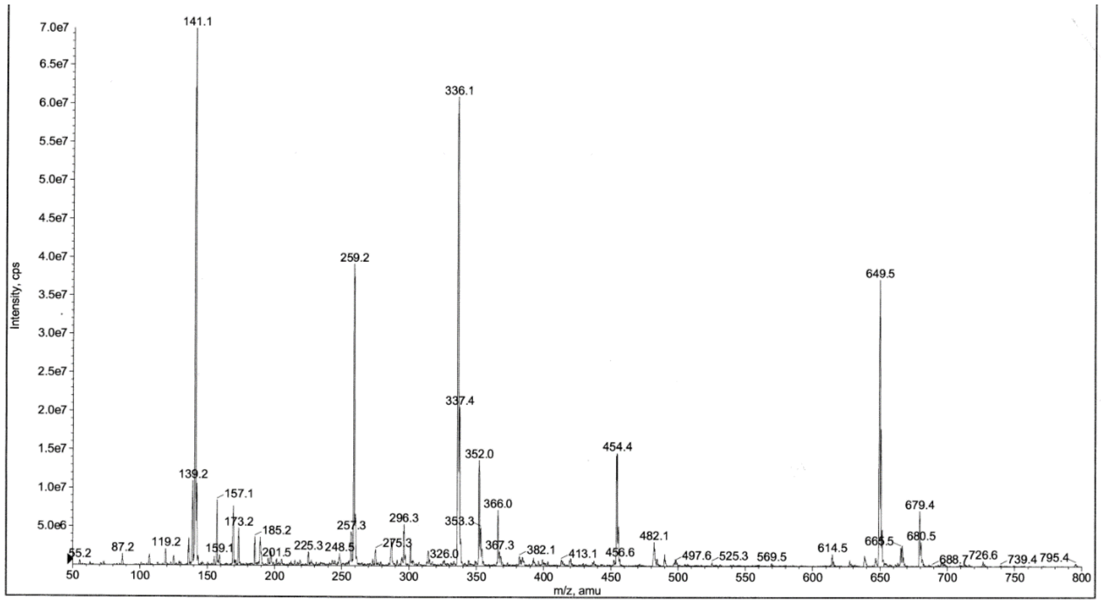

Figure S7. ESI-MS spectrum of the compound **1**

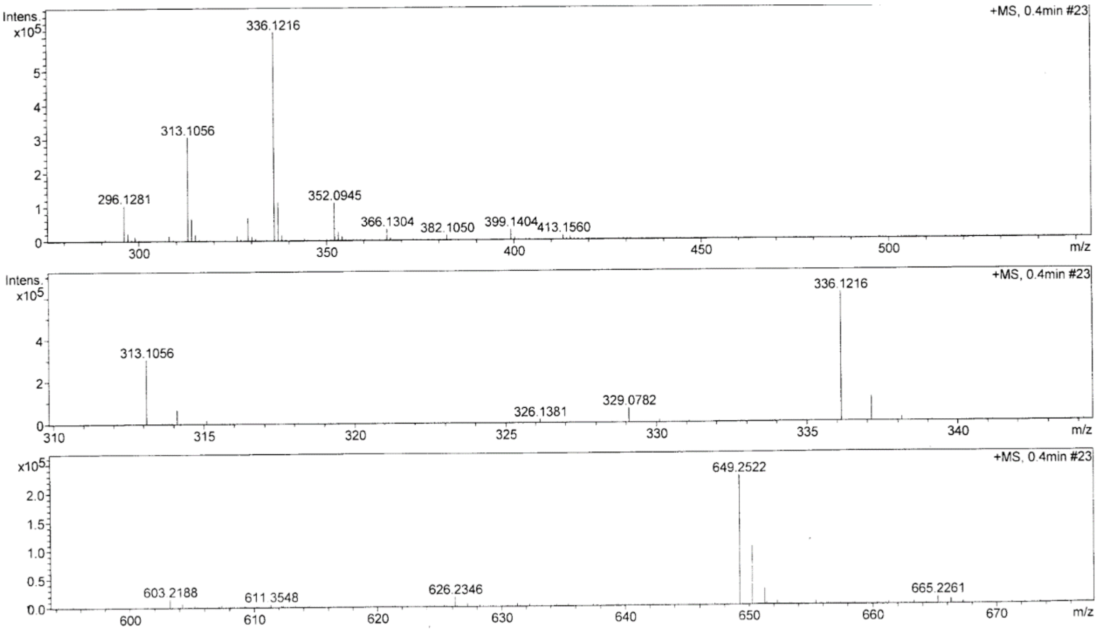

Figure S8. HR-ESI-MS spectrum of the compound **1**

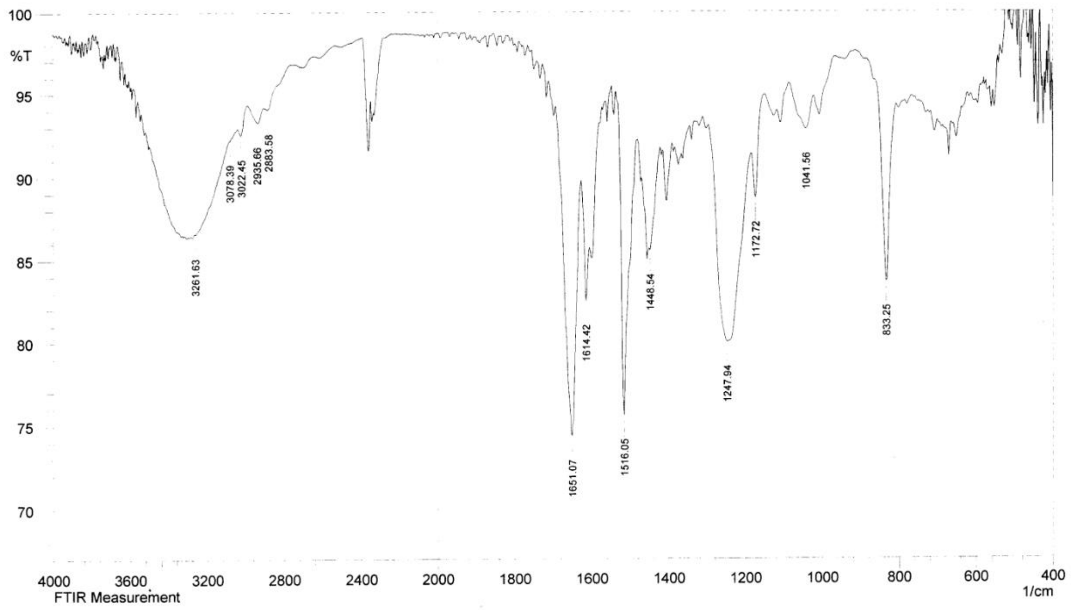

Figure S9. IR spectrum of the compound **1**

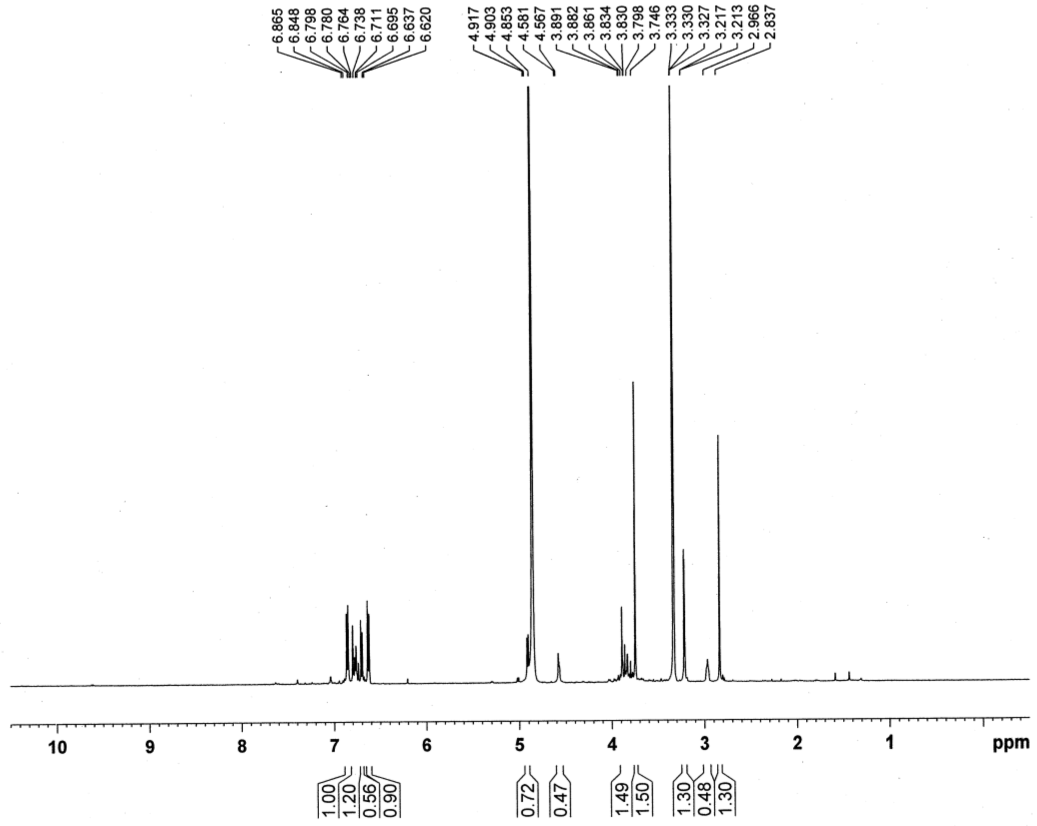

Figure S10.^{1}H-NMR (500MHz, CD_3OD) spectrum of the compound **2**

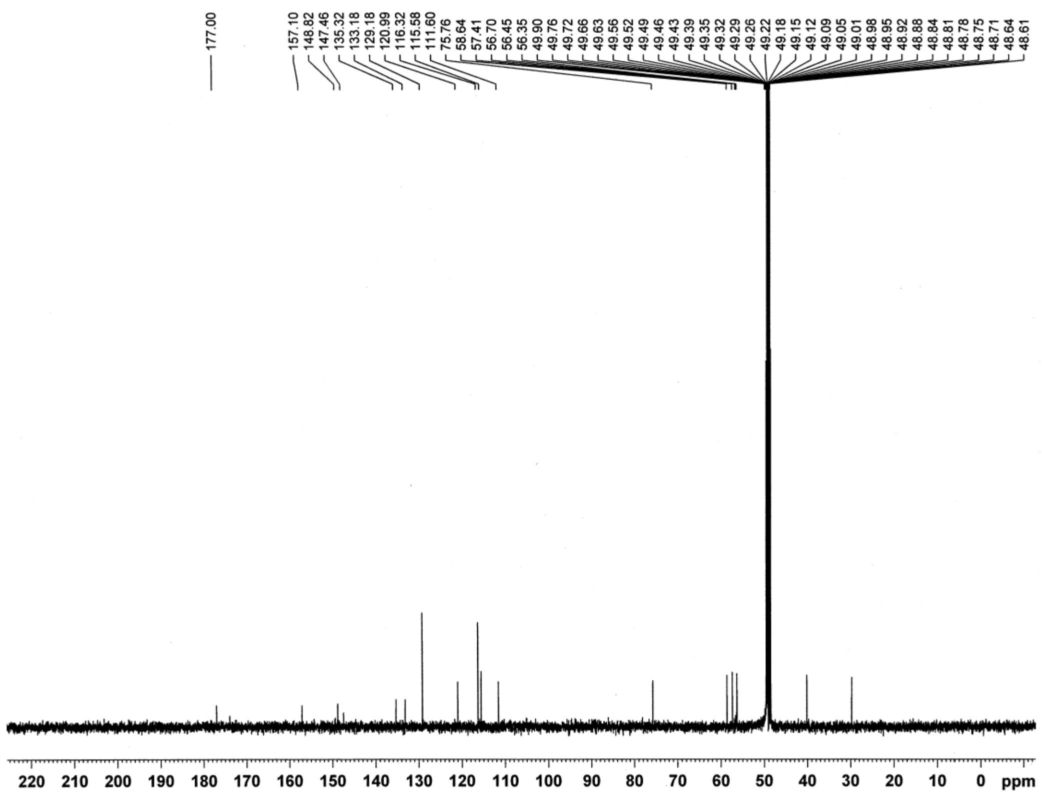

Figure S11.^{13}C-NMR (125MHz, CD_3OD) spectrum of the compound **2**

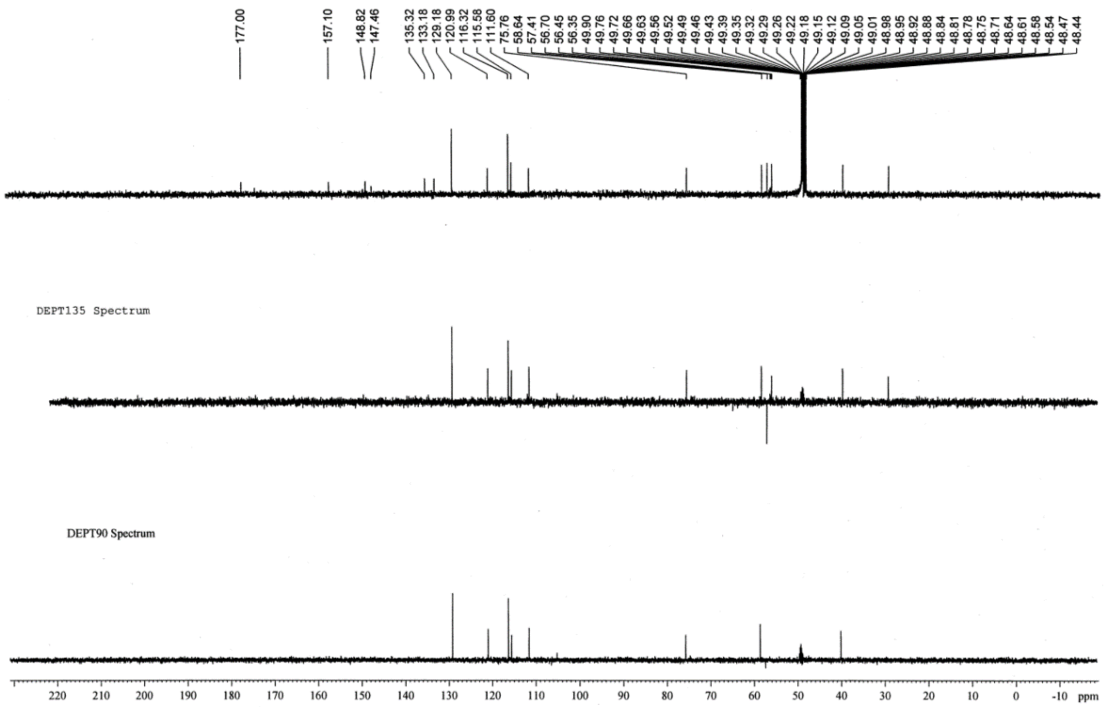

Figure S12. DEPT spectrum of the compound **2**

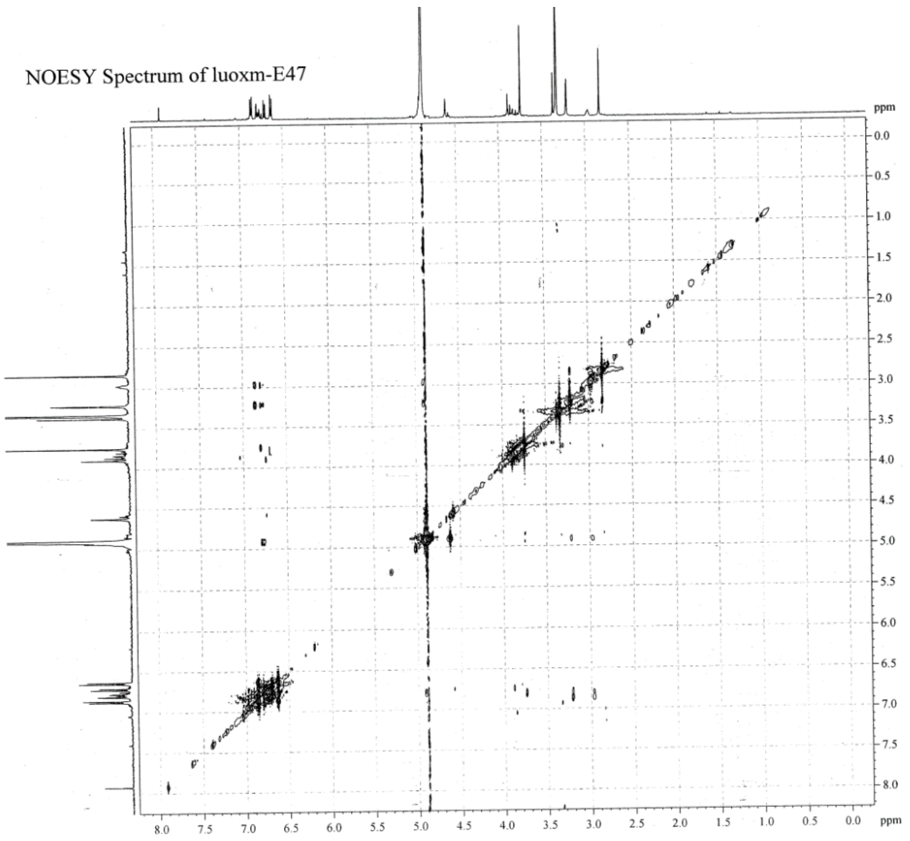

Figure S13.NOESY spectrum of the compound **2**

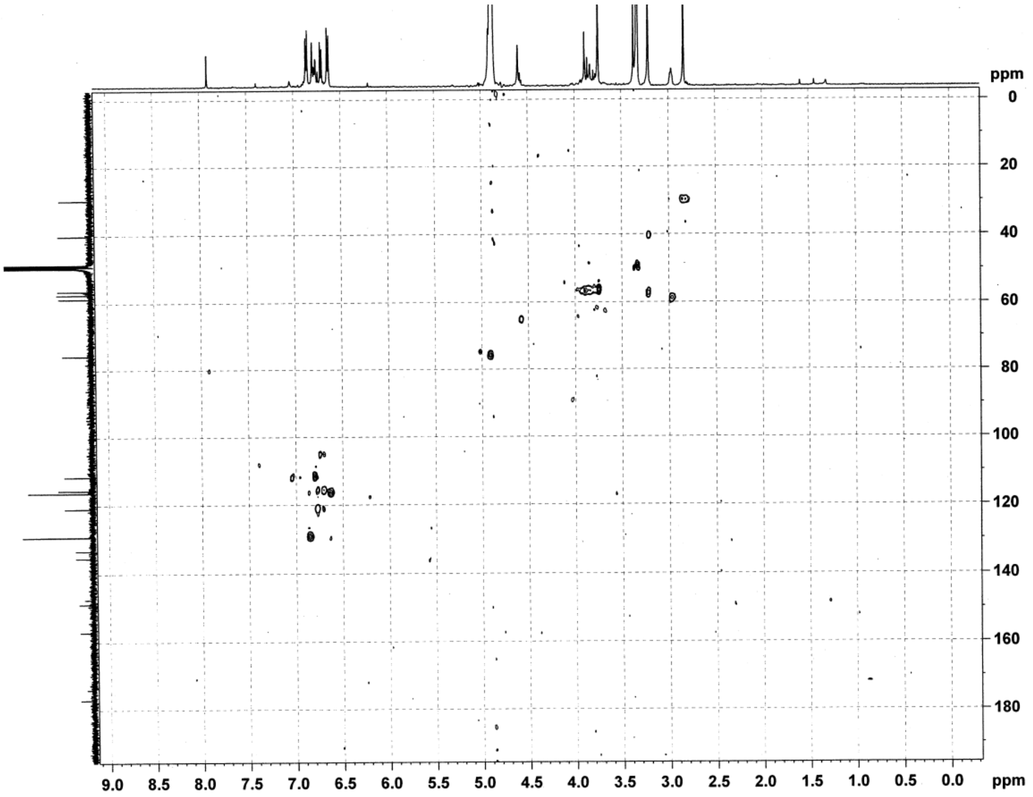

Figure S14. HSQC spectrum of the compound **2**

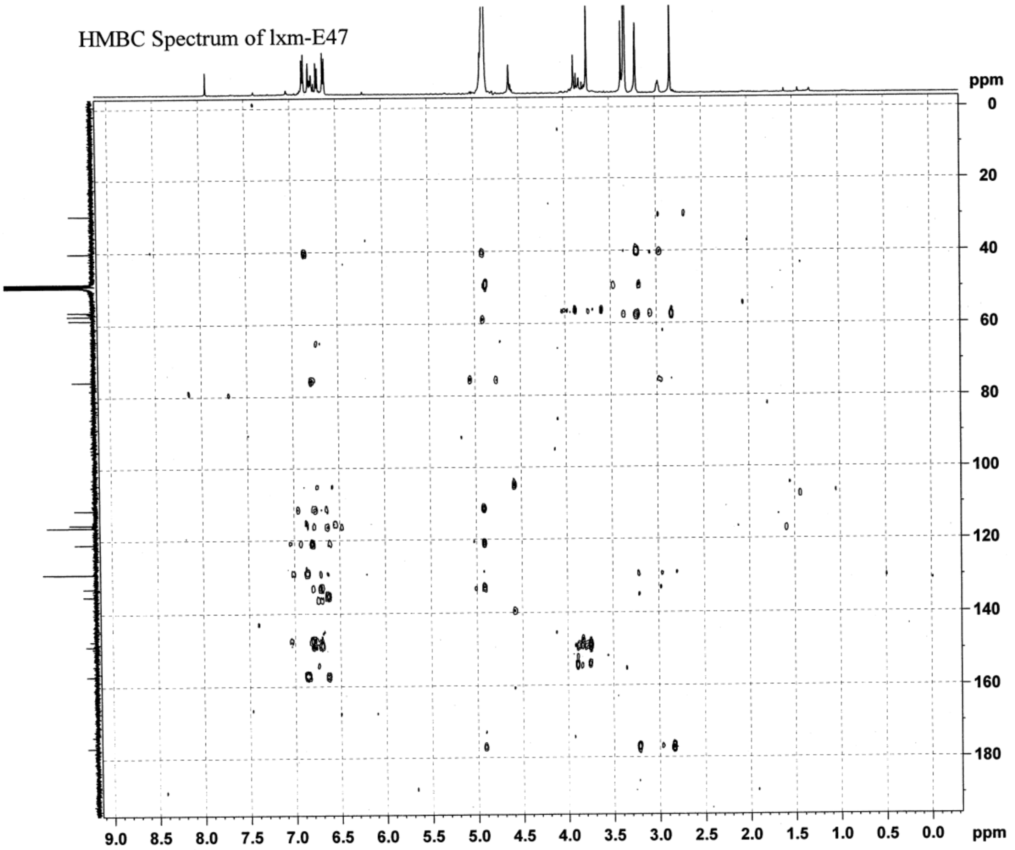

Figure S15. HMBC spectrum of the compound **2**

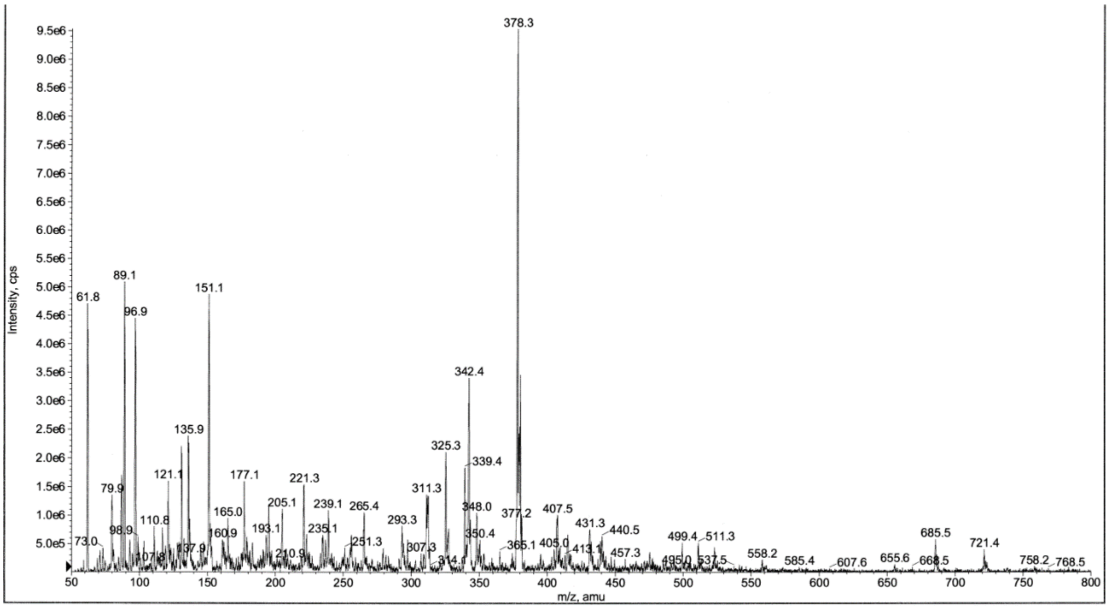

Figure S16. ESI-MS spectrum of the compound **2**

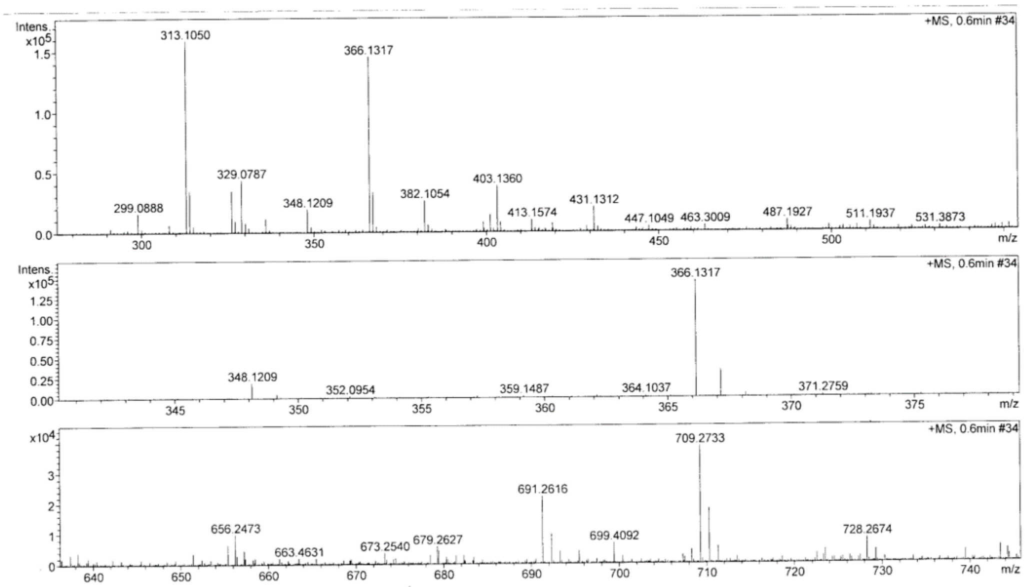

Figure S17. HR-ESI-MS spectrum of the compound **2**

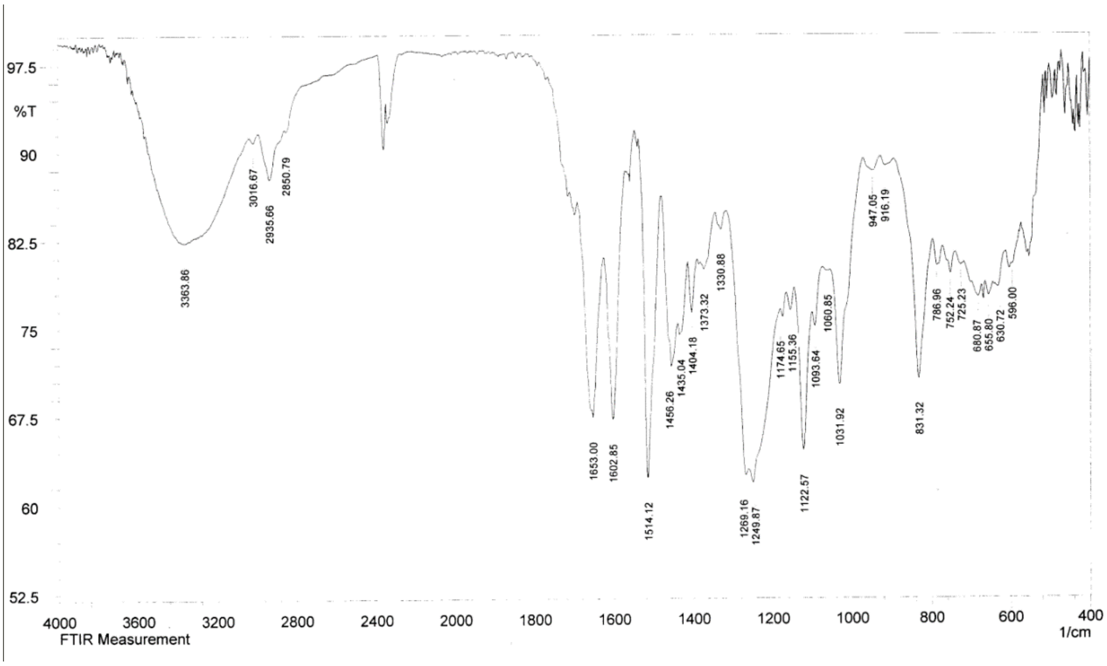

Figure S18. IR spectrum of the compound **2**

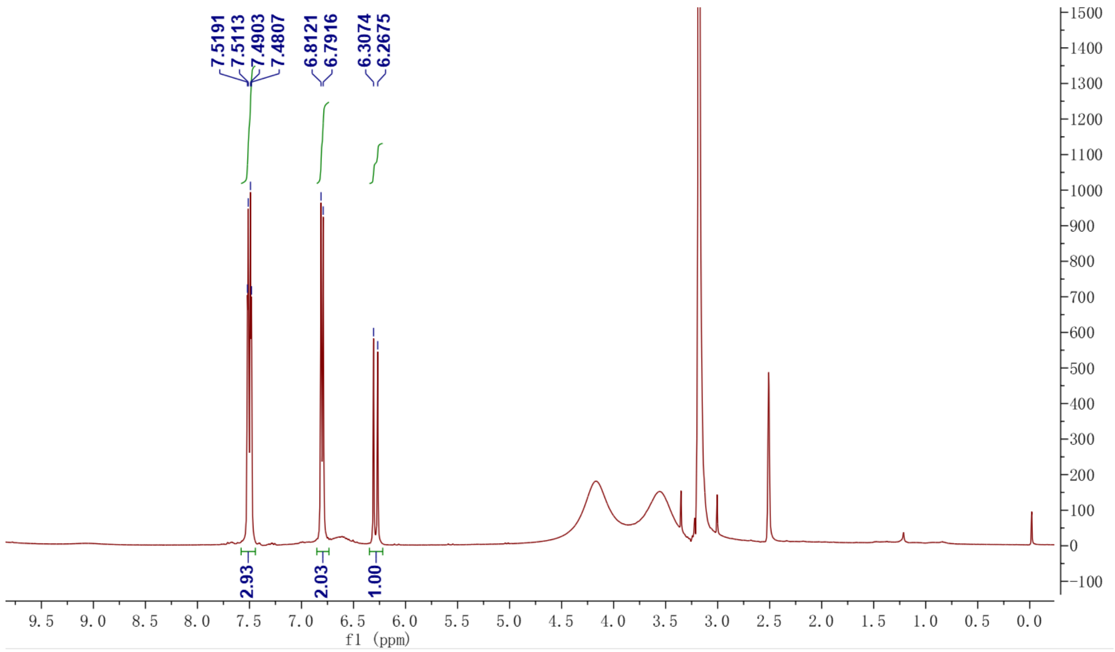

Figure S19.^{1}H-NMR (400MHz, DMSO-d_6) spectrum of the compound **3**

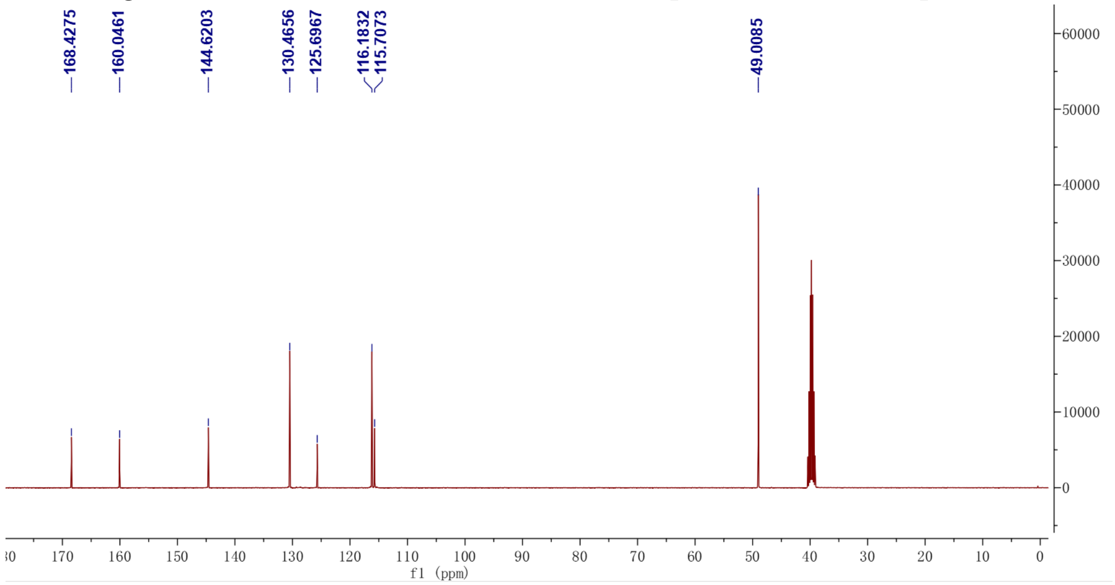

Figure S20.^{13}C-NMR (100MHz, DMSO-d_6) spectrum of the compound **3**

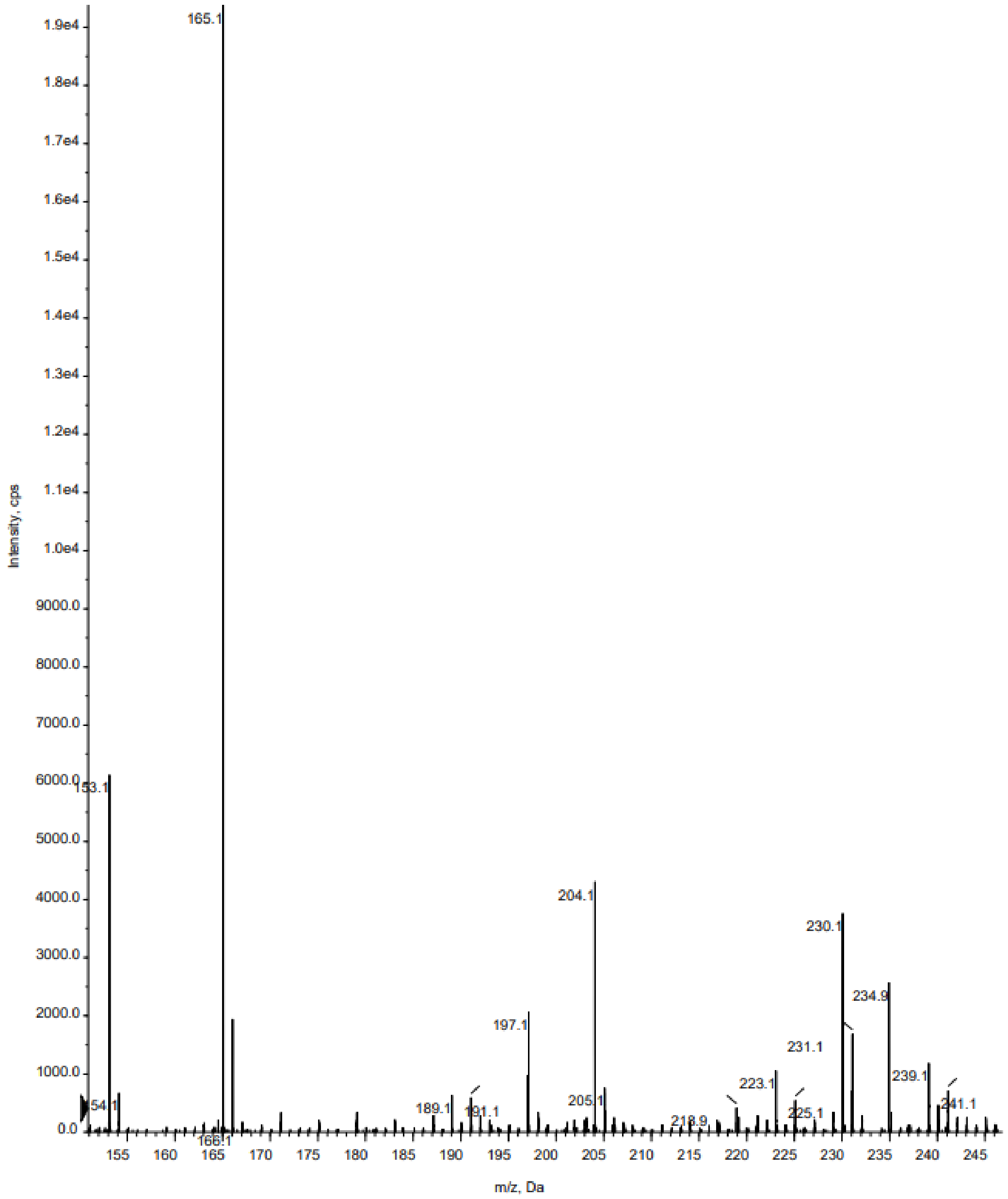

Figure S21. ESI-MS spectrum of the compound **3**

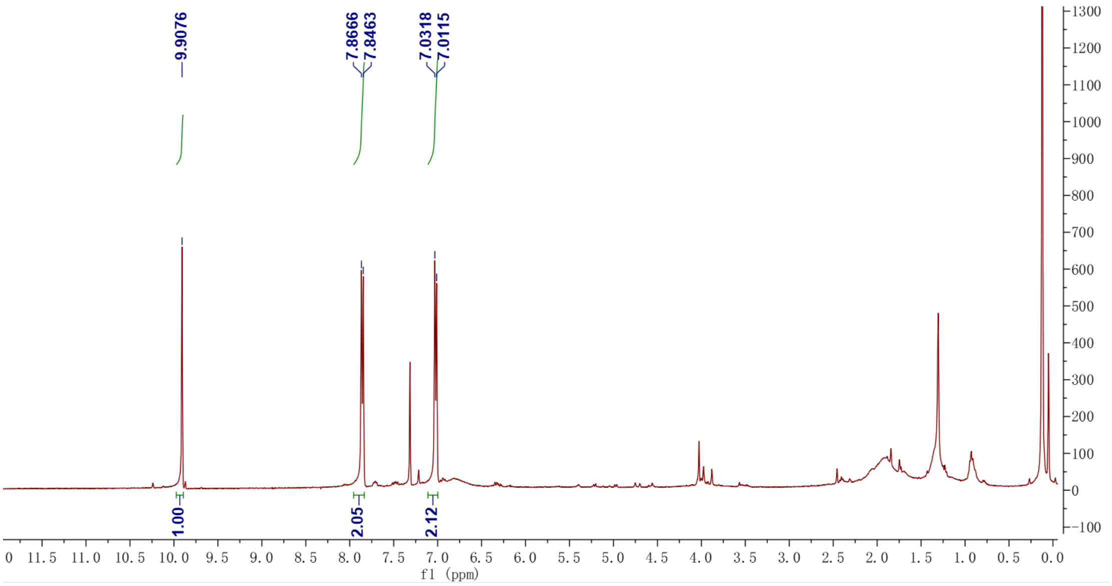

Figure S22.^{1}H-NMR (400MHz, $CDCl_3$) spectrum of the compound **4**

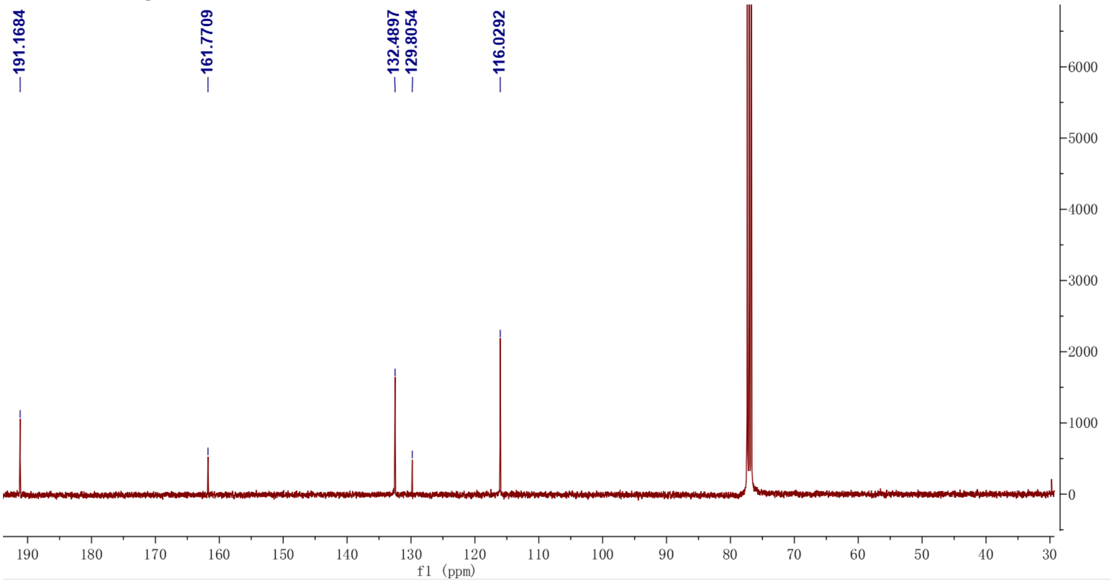

Figure S23.^{13}C-NMR (100MHz, $CDCl_3$) spectrum of the compound **4**

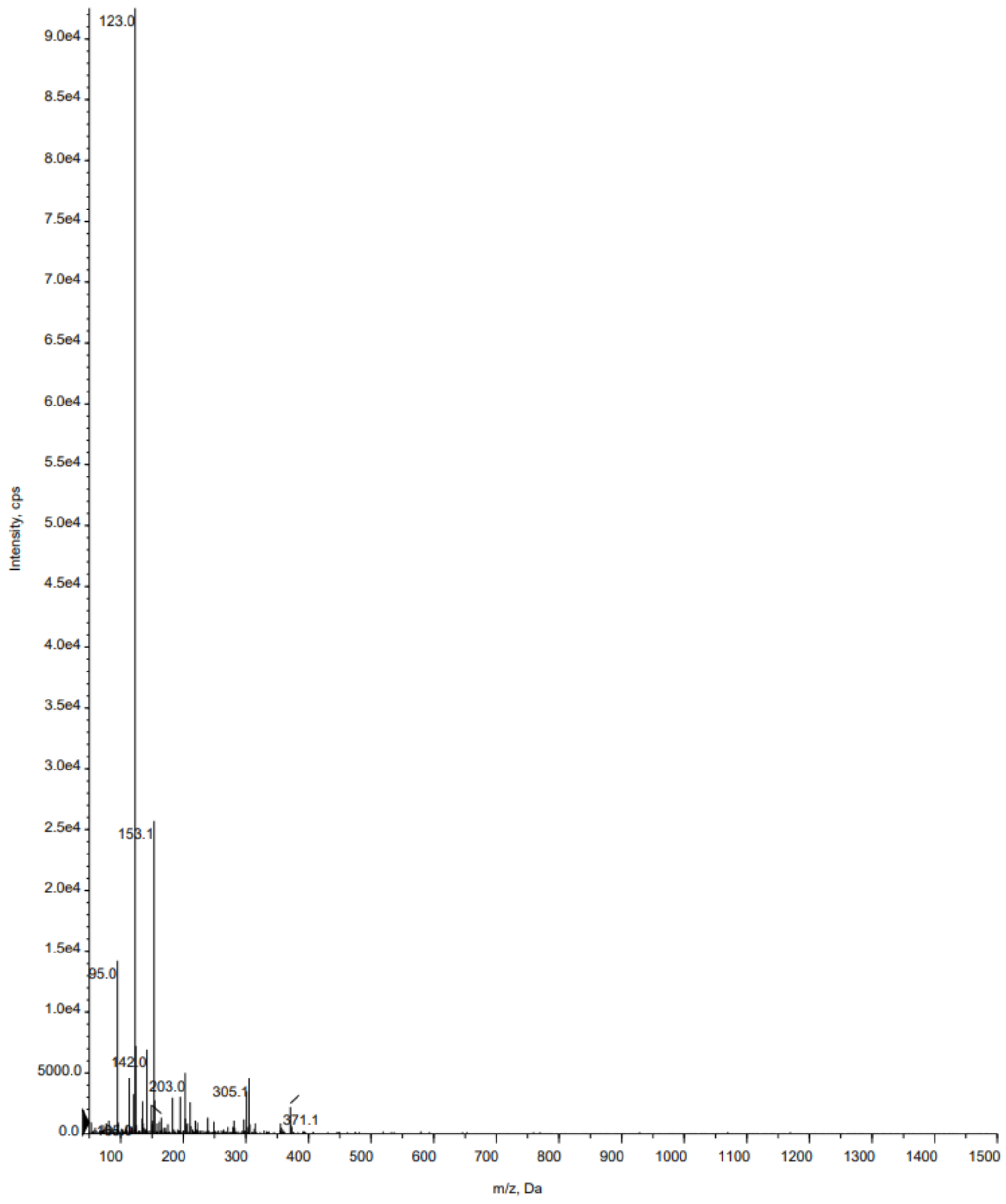

Figure S24. ESI-MS spectrum of the compound **4**

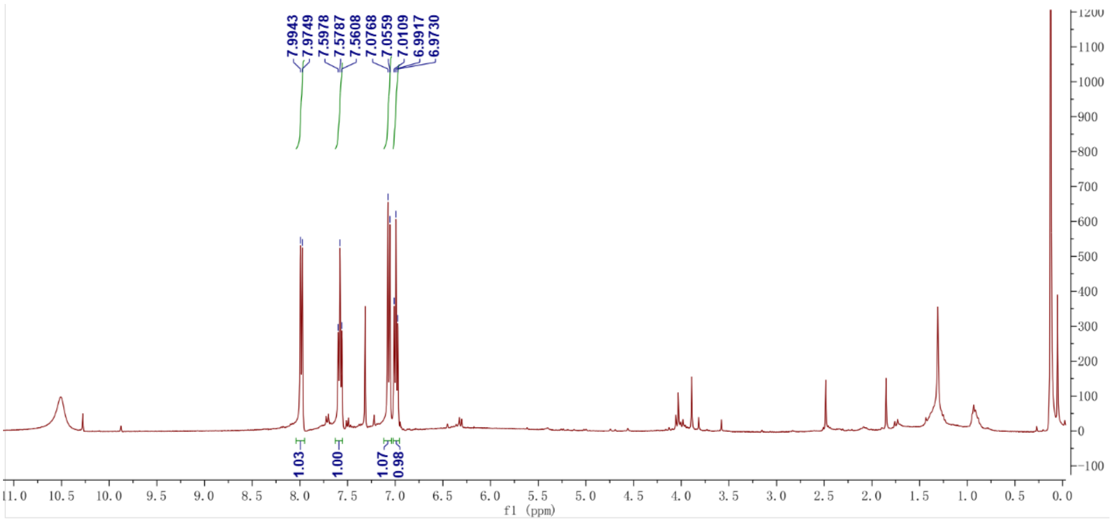

Figure S25. ^{1}H-NMR (400MHz, $CDCl_3$) spectrum of the compound **5**

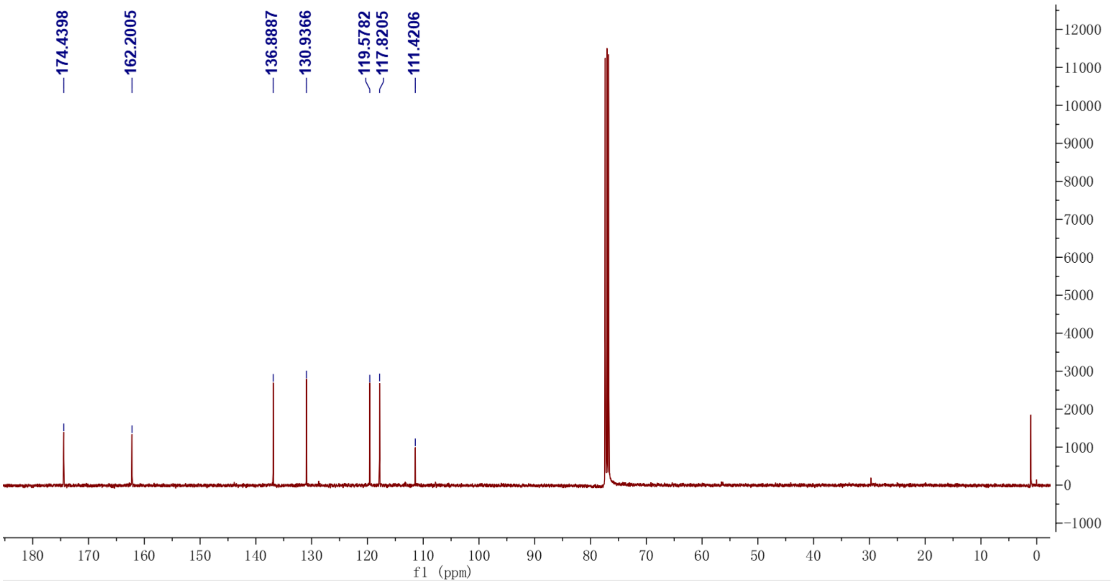

Figure S26. ^{13}C-NMR (100MHz, $CDCl_3$) spectrum of the compound **5**

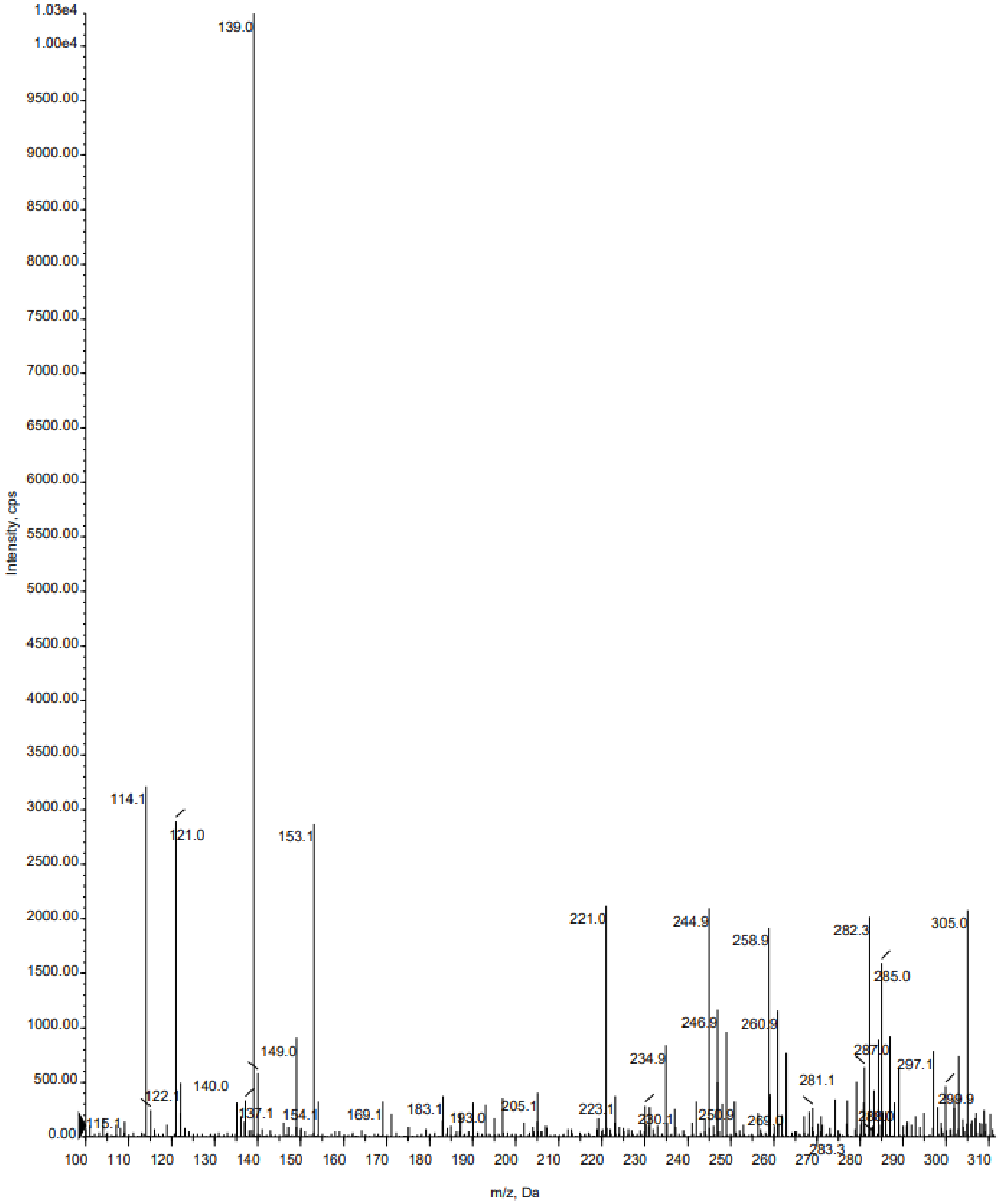

Figure S27. ESI-MS spectrum of the compound **5**

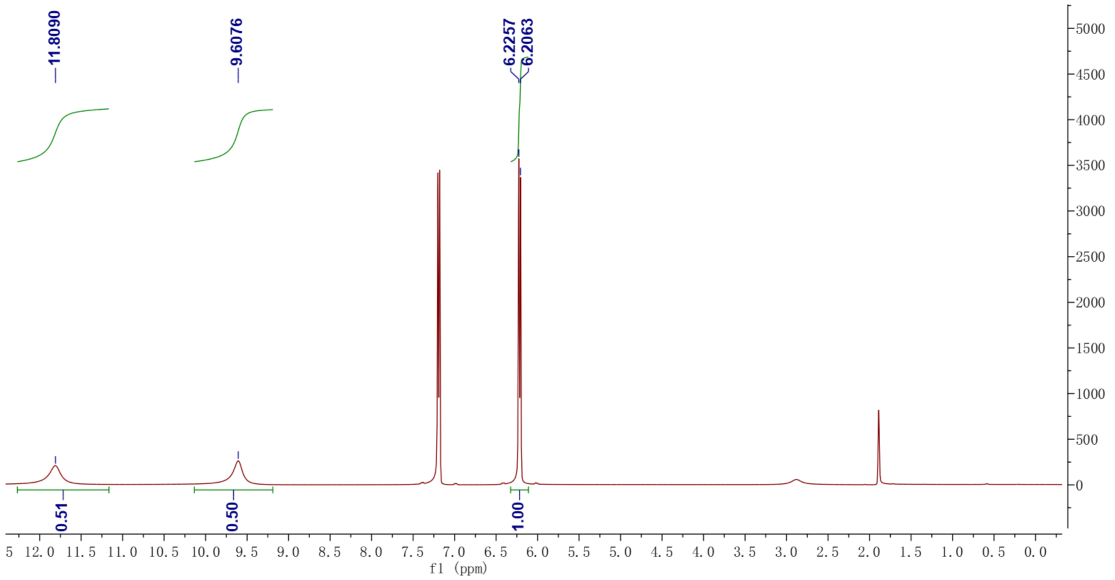

Figure S28.^{1}H-NMR (400MHz, $CDCl_3$) spectrum of the compound **6**

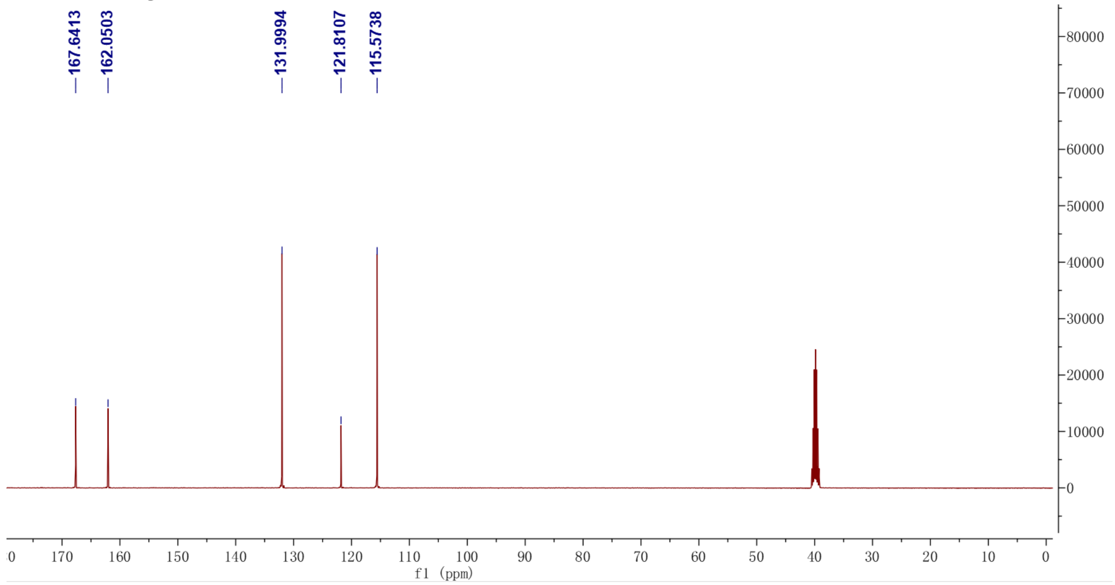

Figure S29.^{13}C-NMR (100MHz, $CDCl_3$) spectrum of the compound **6**

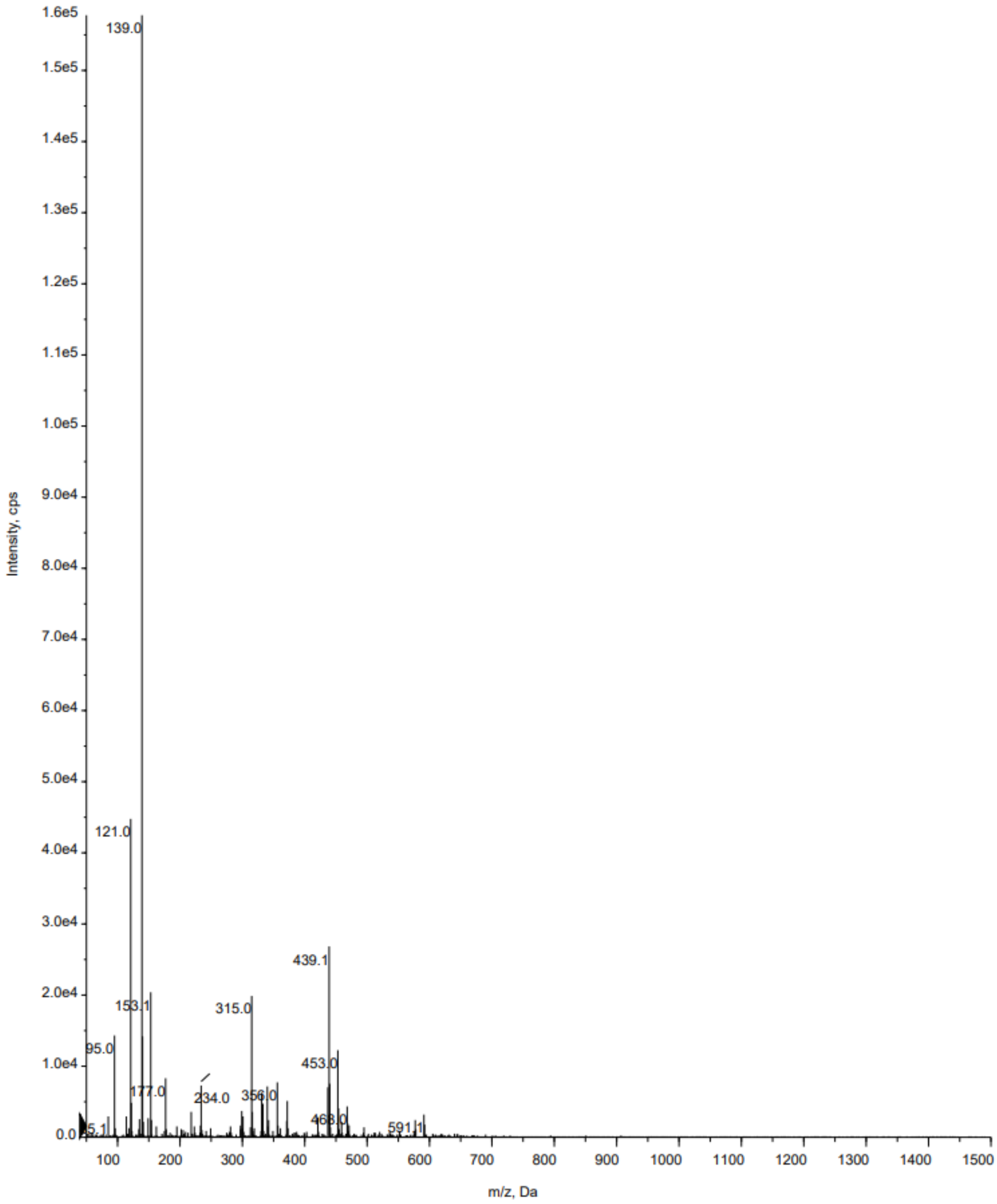

Figure S30. ESI-MS spectrum of the compound **6**

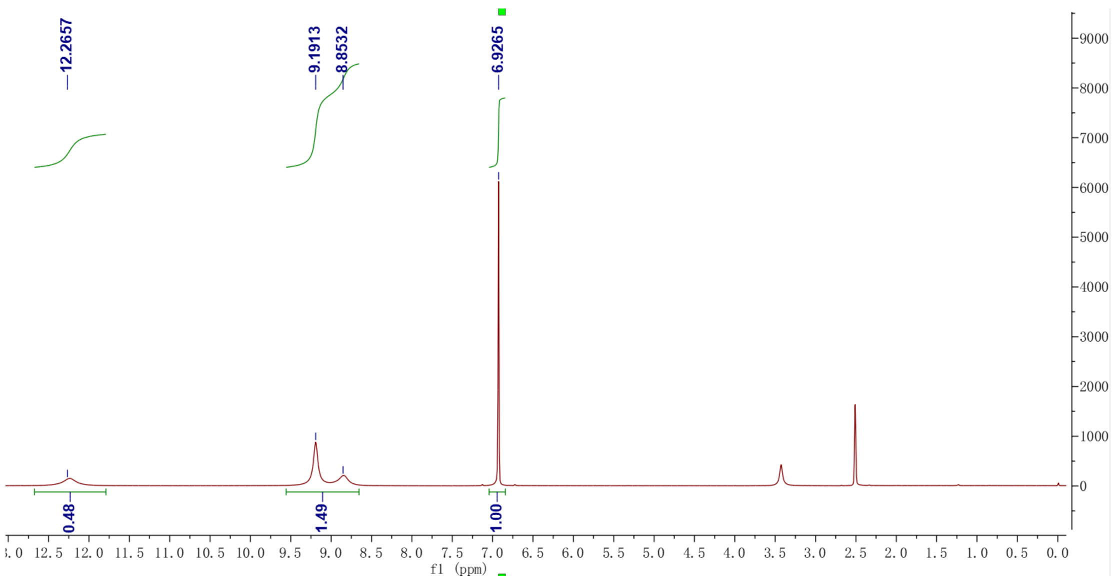

Figure S31.^{1}H-NMR (400MHz, DMSO-d6) spectrum of the compound **7**

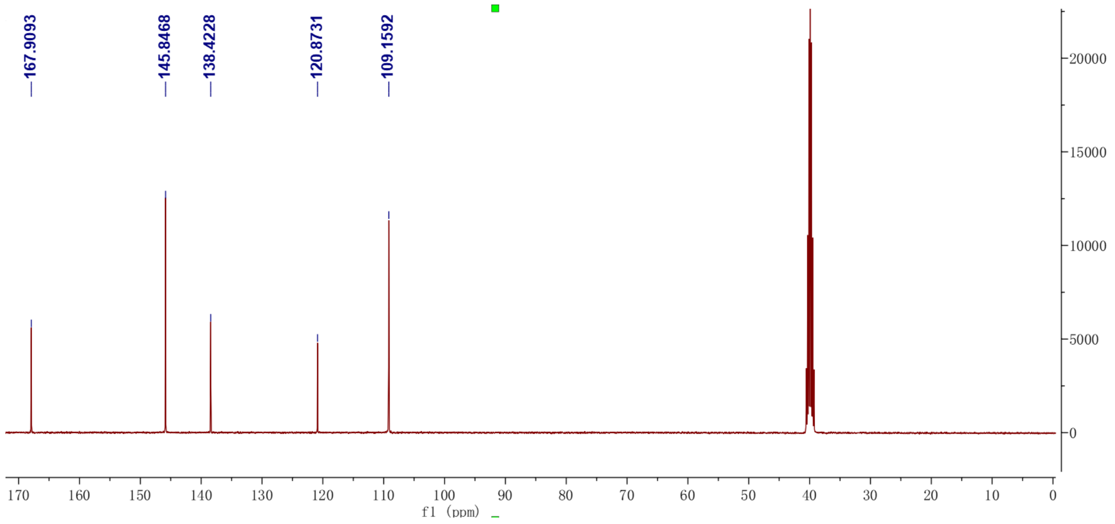

Figure S32.^{13}C-NMR (100MHz, DMSO-d6) spectrum of the compound **7**

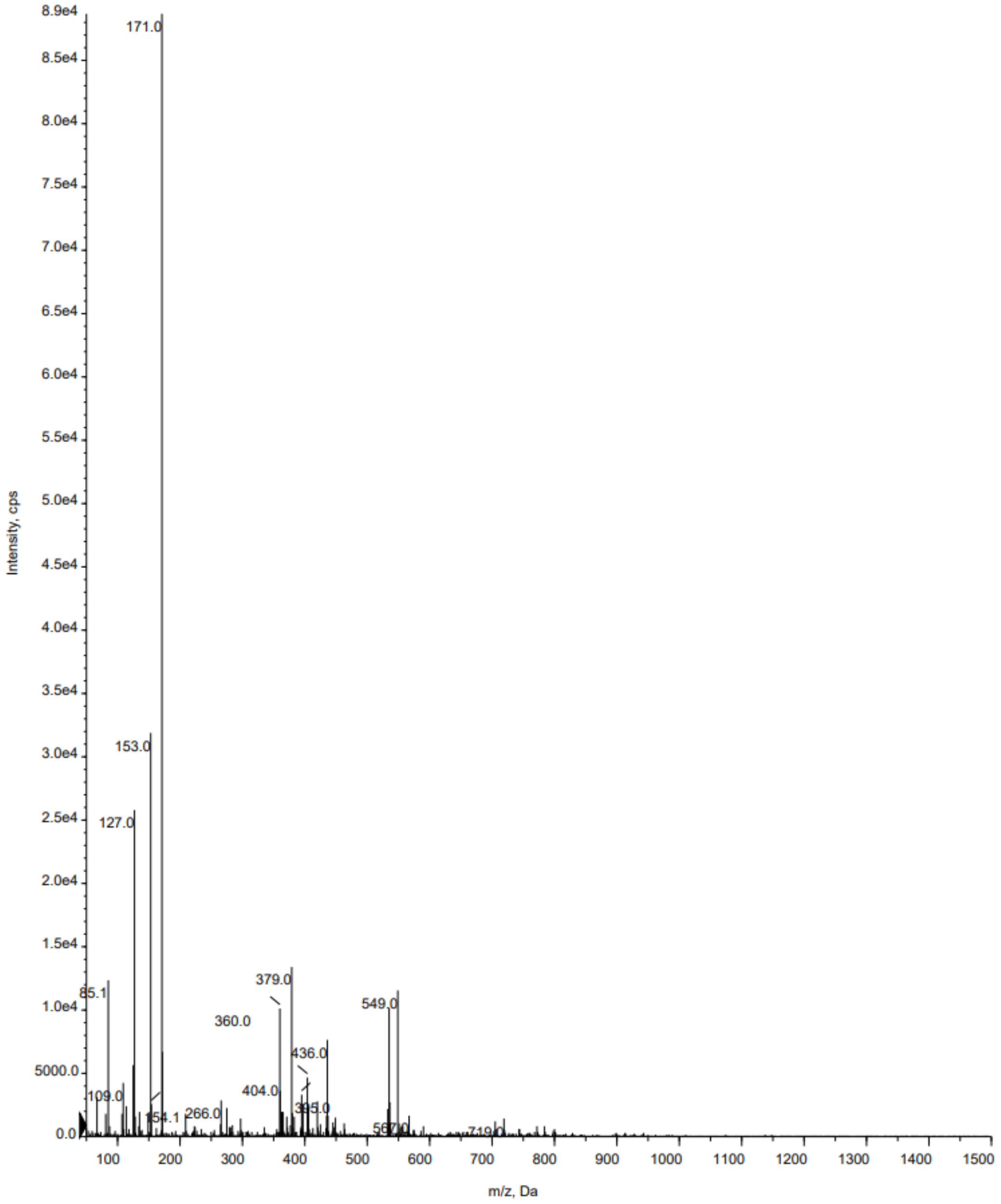

Figure S33. ESI-MS spectrum of the compound **7**
